# Supplementary material for: Medication and atypical brain maturation in psychosis are associated with long-term cognitive decline and symptom progression
Source: Br J Psychiatry. Author manuscript; Available in PMC 2025 Dec 17. (PMC7618483; doi:10.1192/bjp.2025.10482)
Supplement: Supplementary Material [file EMS210217-supplement-Supplementary_Material.pdf]

Medication and atypical brain maturation in psychosis is associated with long-term cognitive decline and symptom progression

Supplementary Material

Contents

Supplementary Methods 2
Subjects 2
Supplementary Figures 3
Supplementary Tables...15
REFERENCES 28

Supplementary Methods

Subjects

PAFIP (Programa de Atención a las Fases Iniciales de Psicosis) participants underwent screening based on the following criteria[1]: (1) age range of 15 to 60 years; (2) meeting DSM-IV criteria for a primary diagnosis of schizophreniform disorder, schizophrenia, schizoaffective disorder, brief reactive psychosis, schizotypal personality disorder, or psychosis not otherwise specified; (3) permanent residency within the designated catchment area; (4) absence of prior exposure to antipsychotic medication or, if previously treated, a cumulative lifetime history of sufficient antipsychotic symptoms of at least moderate severity, as evaluated by one of the five items of the Scale for the Assessment of Positive Symptoms. (SAPS).

Patients were ineligible for inclusion in the study if they met any of the following criteria: 1) met DSM-IV criteria for drug dependence (excluding nicotine dependence), 2) met DSM-IV criteria for mental retardation, or 3) had a history of neurological disease or head injury. There were no additional exclusion criteria for MRI scans except those related to logistical constraints (e.g., claustrophobia, braces). The control group comprising healthy volunteers was recruited from the community via advertisements. They were required to have no current or previous psychiatric disorders, intellectual disability , neurological conditions, or significant medical illnesses, including substance dependence and prolonged loss of consciousness, as assessed using a condensed version of the Comprehensive Assessment of Symptoms and History [2]. Selection criteria aimed to achieve similar distributions in age, gender, and drug use history as the patient group. Furthermore, clinical records and family interviews were utilized to verify the absence of psychosis in first-degree relatives. Following a comprehensive explanation of the study, each participant provided written informed consent prior to enrolment.

Supplementary Figures

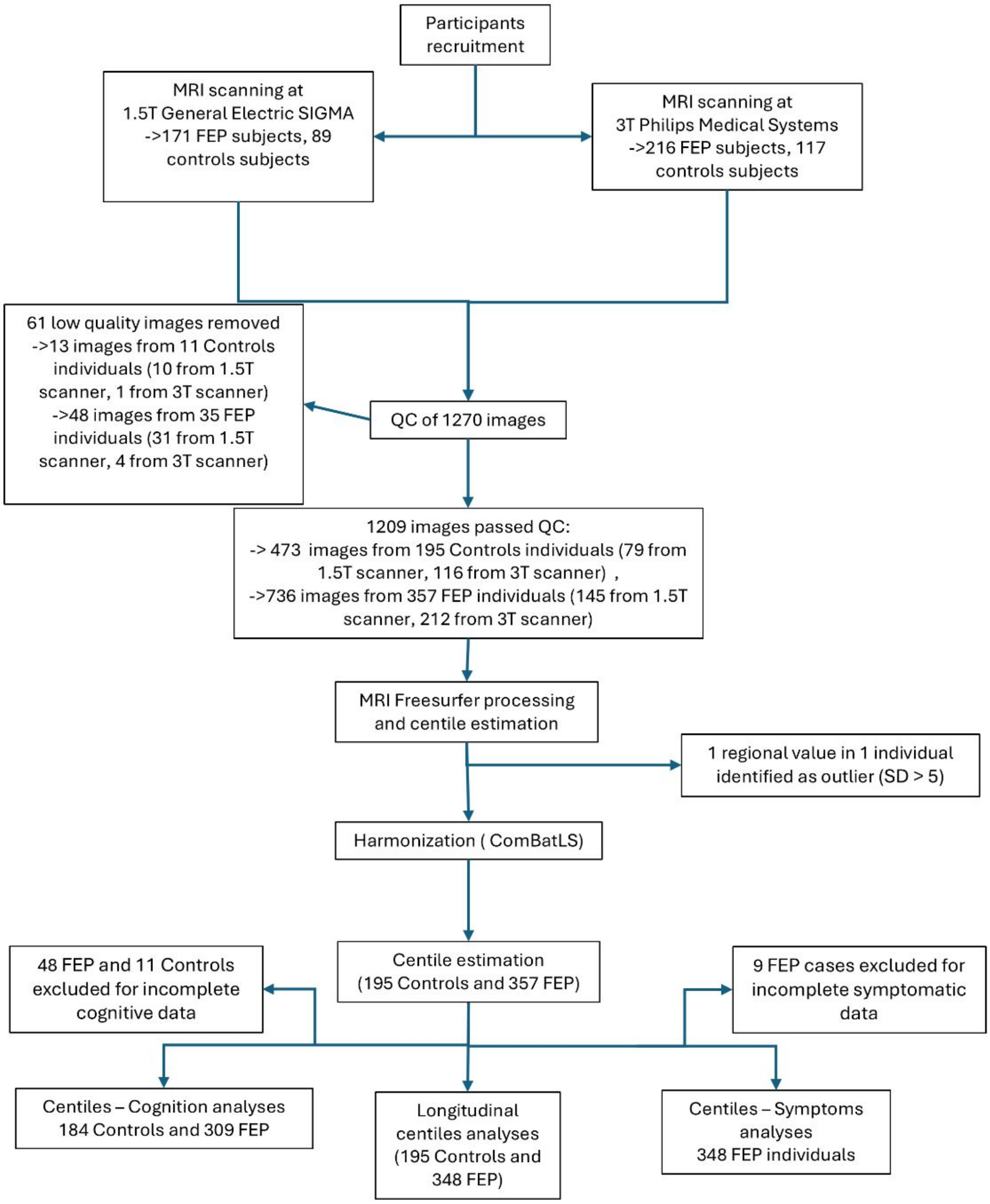

Fig. S1. Flow diagram for study participants.

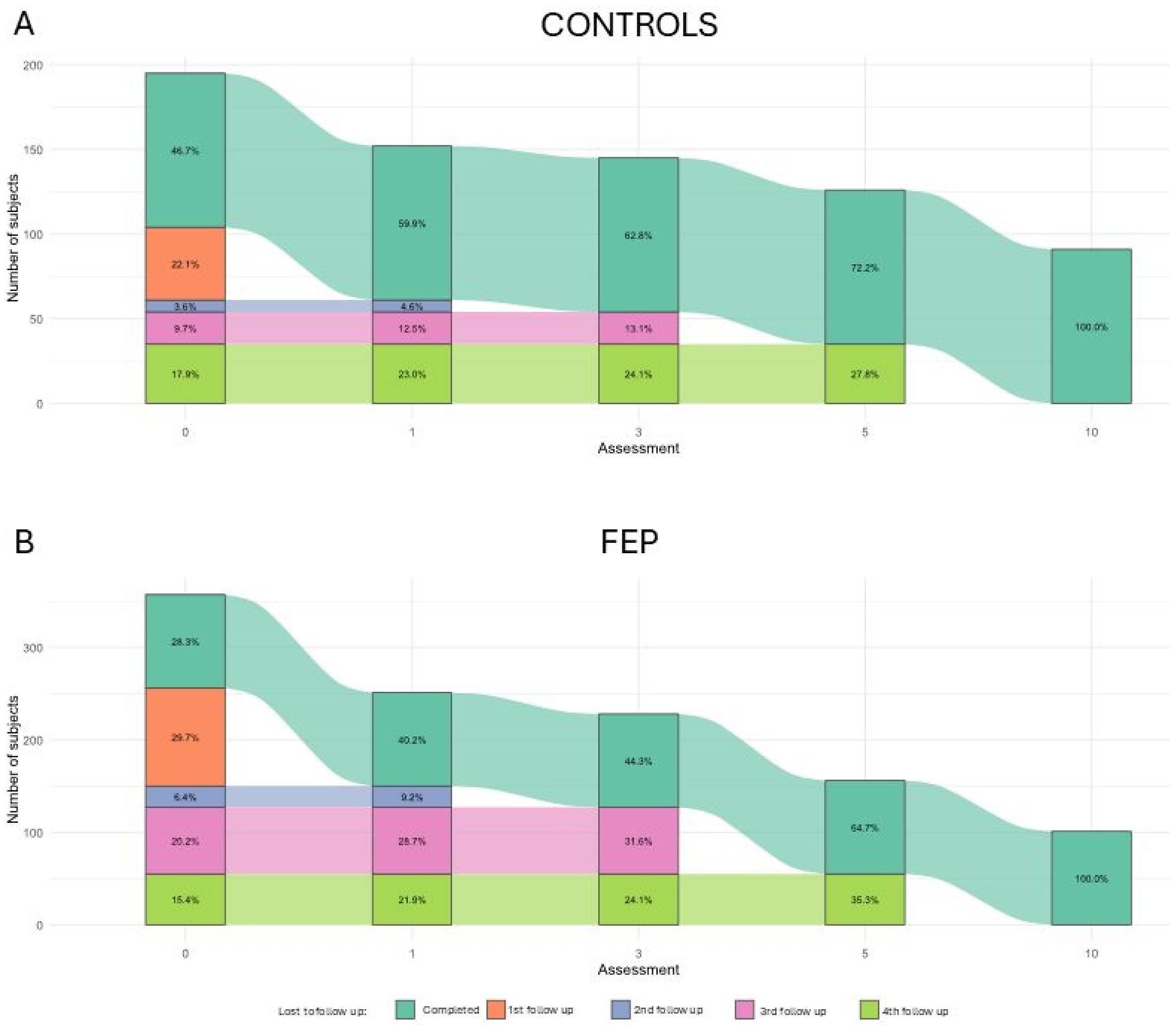

Fig. S2. Alluvial diagram illustrating participant attrition.

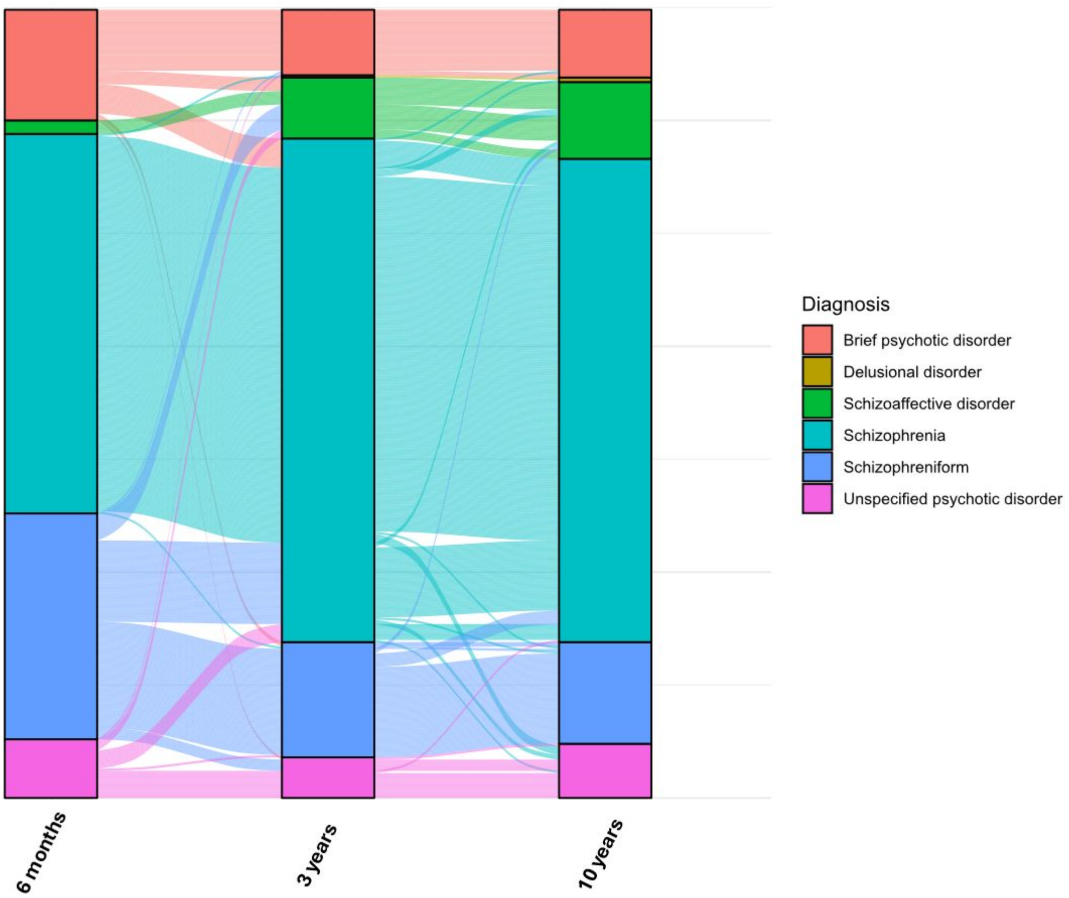

Fig. S3. Alluvial plot depicting participant transitions across diagnostic categories over three assessment timepoints. The plot shows stacked bars (strata) representing the distribution of diagnoses at each timepoint and connecting flows (alluvia) indicating the proportion of participants transitioning between diagnostic categories. The height of each stratum and the width of each flow are proportional to the ratio of FEP participants with each diagnosis.

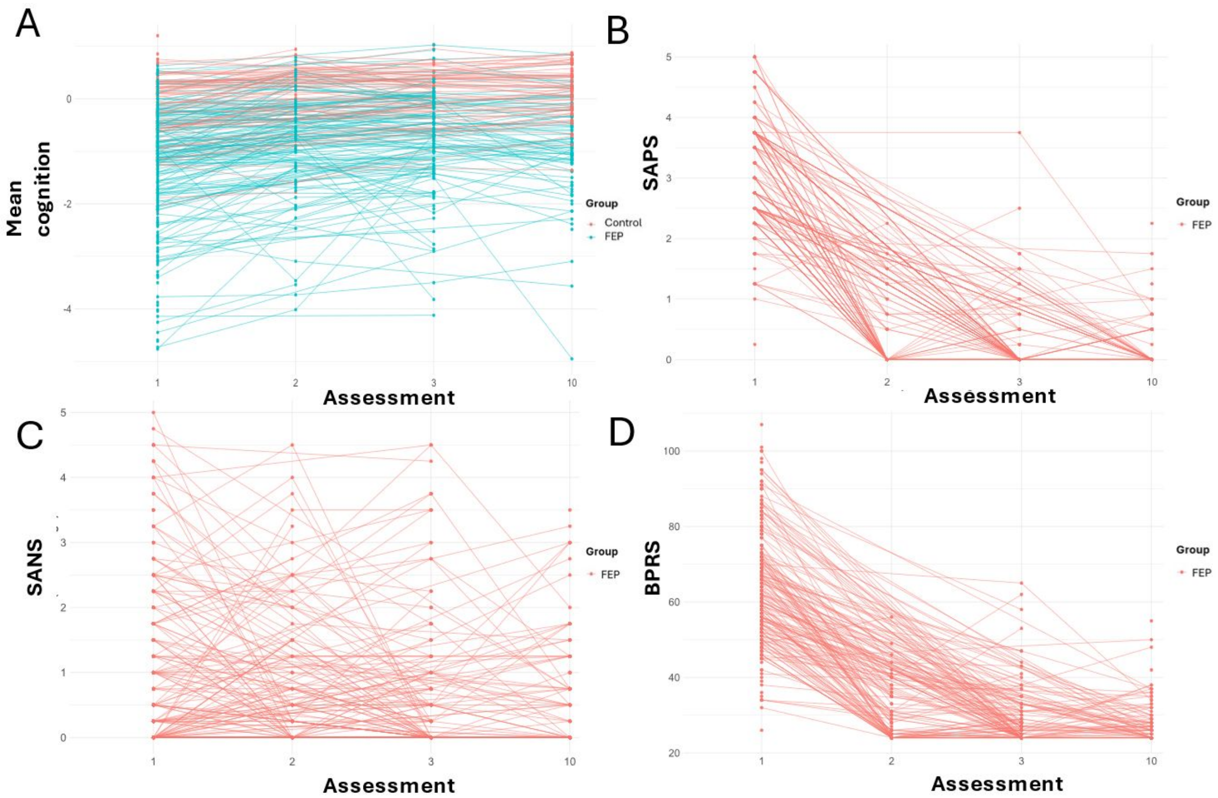

Fig. S4. Longitudinal progression of cognition and symptoms in the cohort.

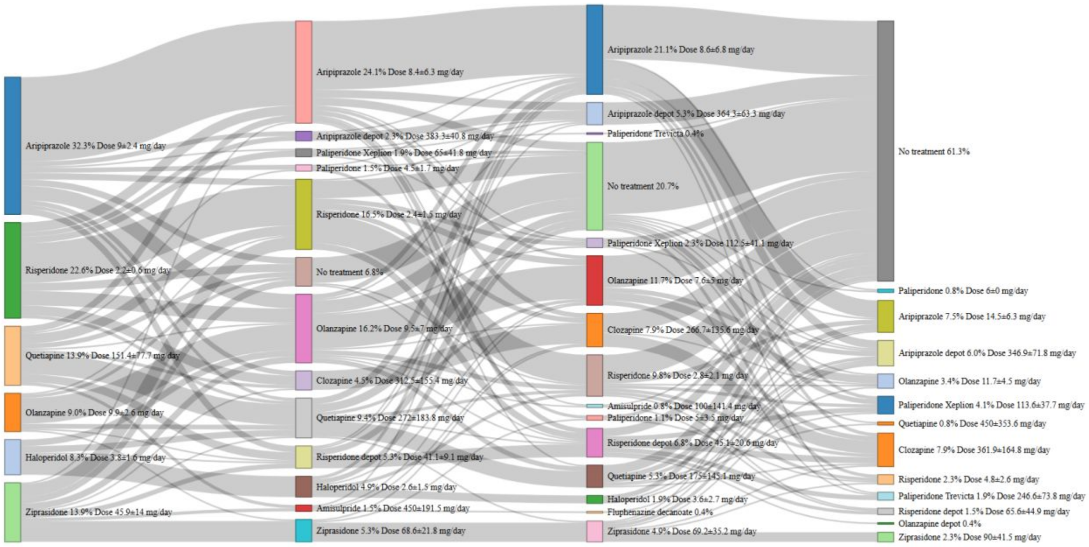

Fig. S5. Alluvial diagram illustrating changes in medication over the course of treatment.

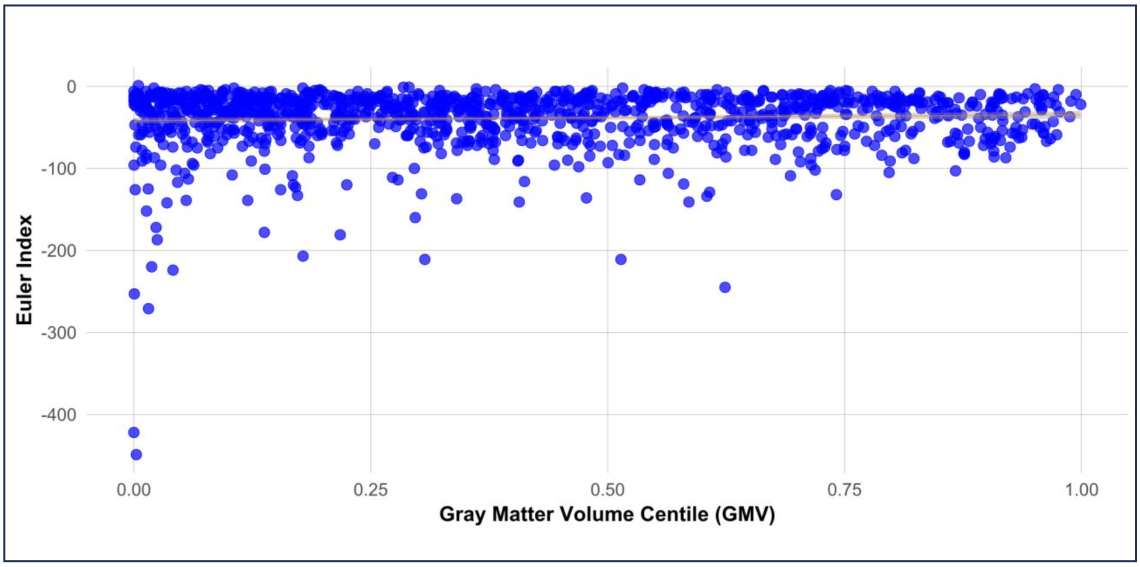

Fig. S6. Relationship between the Euler index of each image and total gray matter centile volume.

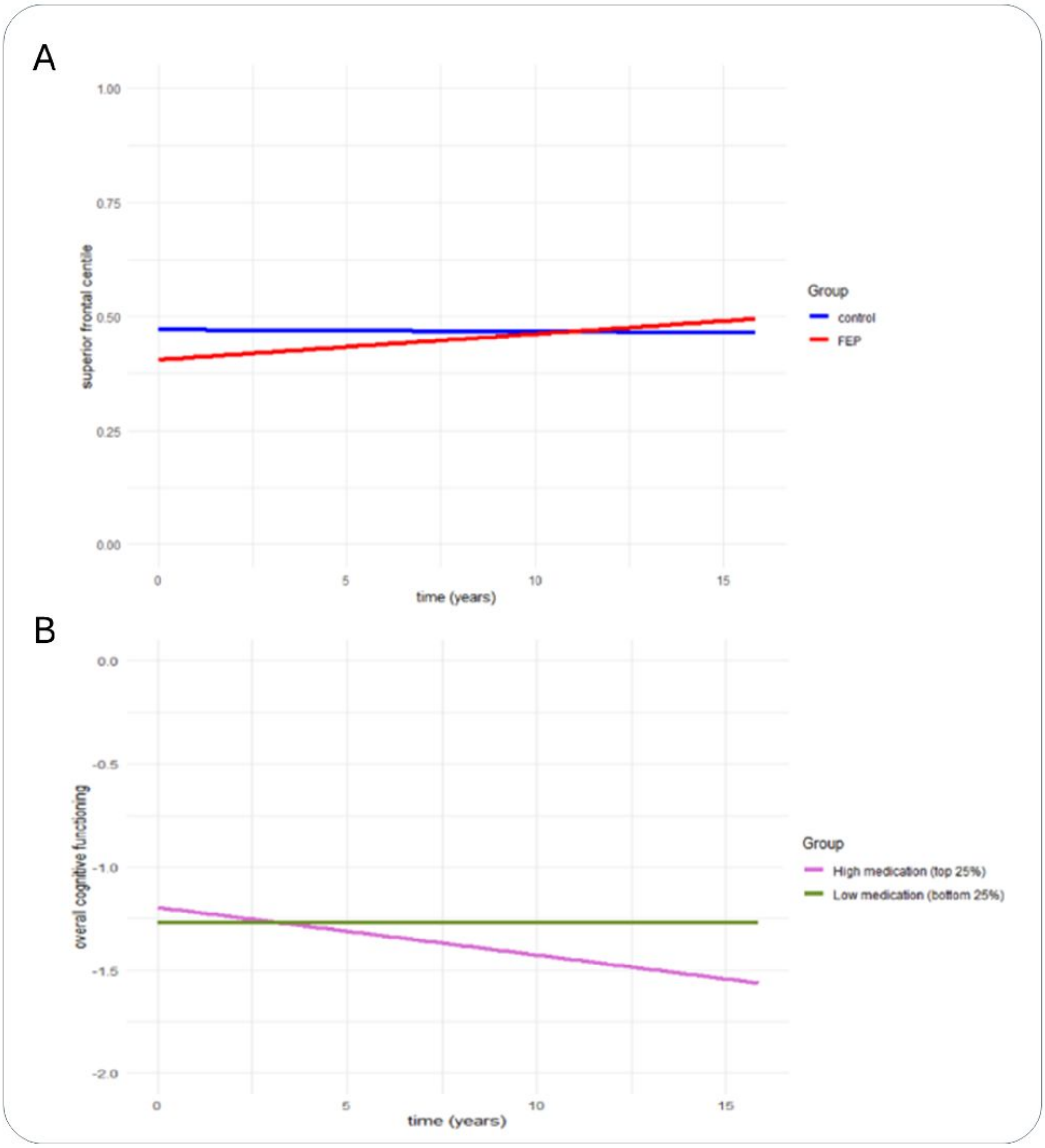

Fig. S7. Illustrative representation of interaction effects between variables in a representative centile. (A) Relationship between superior frontal centile and treatment duration, stratified by diagnosis. (B) Relationship between overall cognitive functioning and treatment duration, stratified by medication.

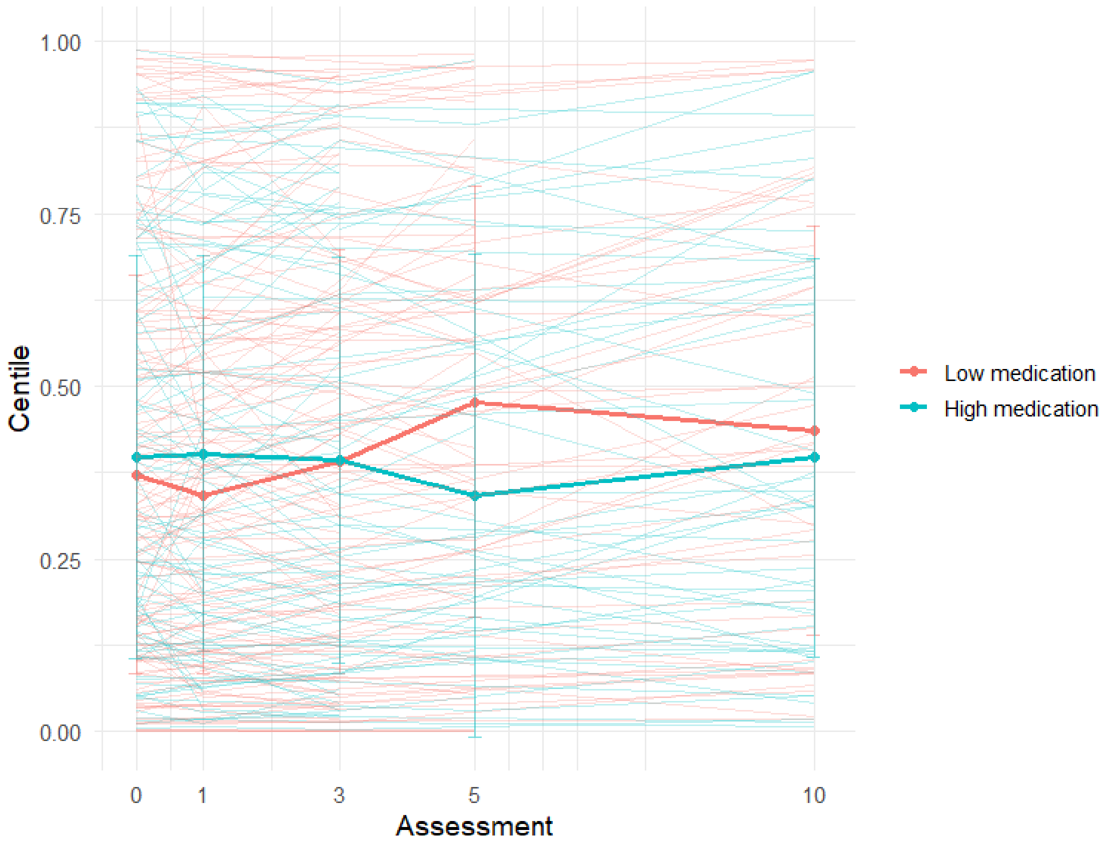

Fig. S8. Global centile across assessments color-coded by patients' medication status. Patients were classified as having high or low medication depending on whether their CPZ-equivalent dosage exceeded 300 at any point during treatment.

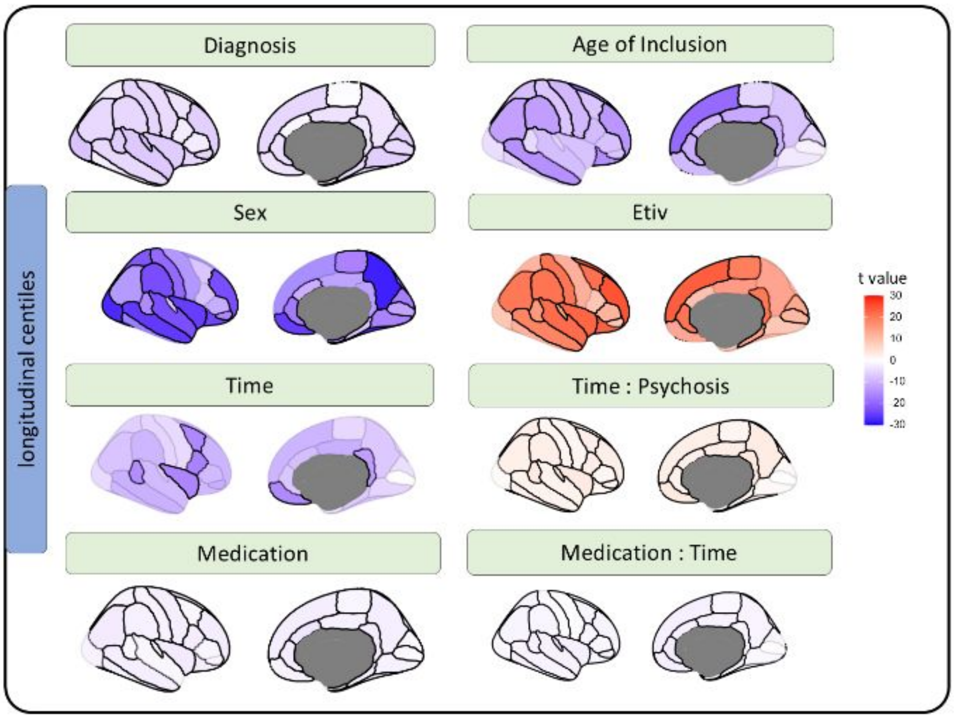

Fig. S9. Associations between clinical data and covariates with raw volumes.

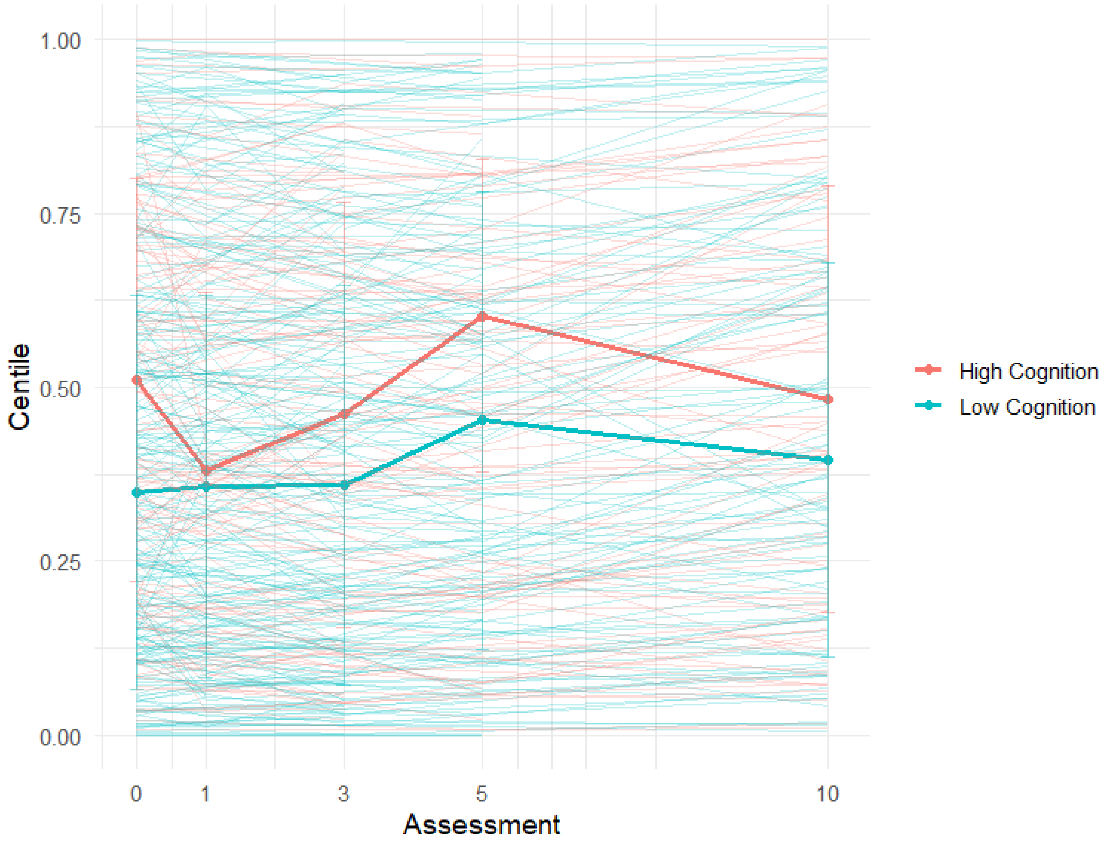

Fig. S10. Global centile across assessments color-coded by patients' final cognitive status, categorized as high or low depending on whether they scored above or below the overall final cognition mean of all study participants.

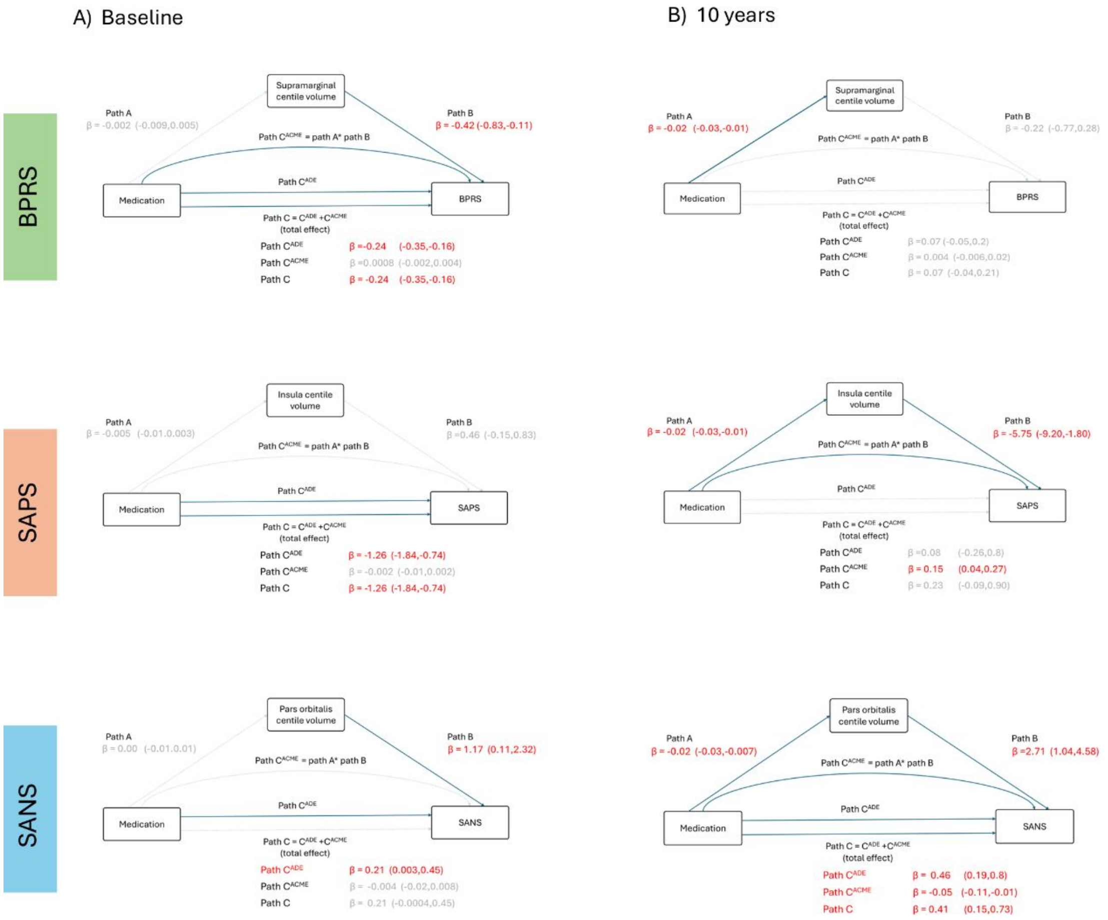

Fig. S11. Mediation analyses of centiles and treatment time on the association between medication and symptomatology. (**A**) Mediation effect at treatment initiation (t=0). (**B**) Mediation effect at 10 years follow-up. Dashed lines show no significant paths. The overall mediation effect of path C is the sum of the average direct effect (path C^{ADE}) and the average causal mediation effect (path C^{ACME}).

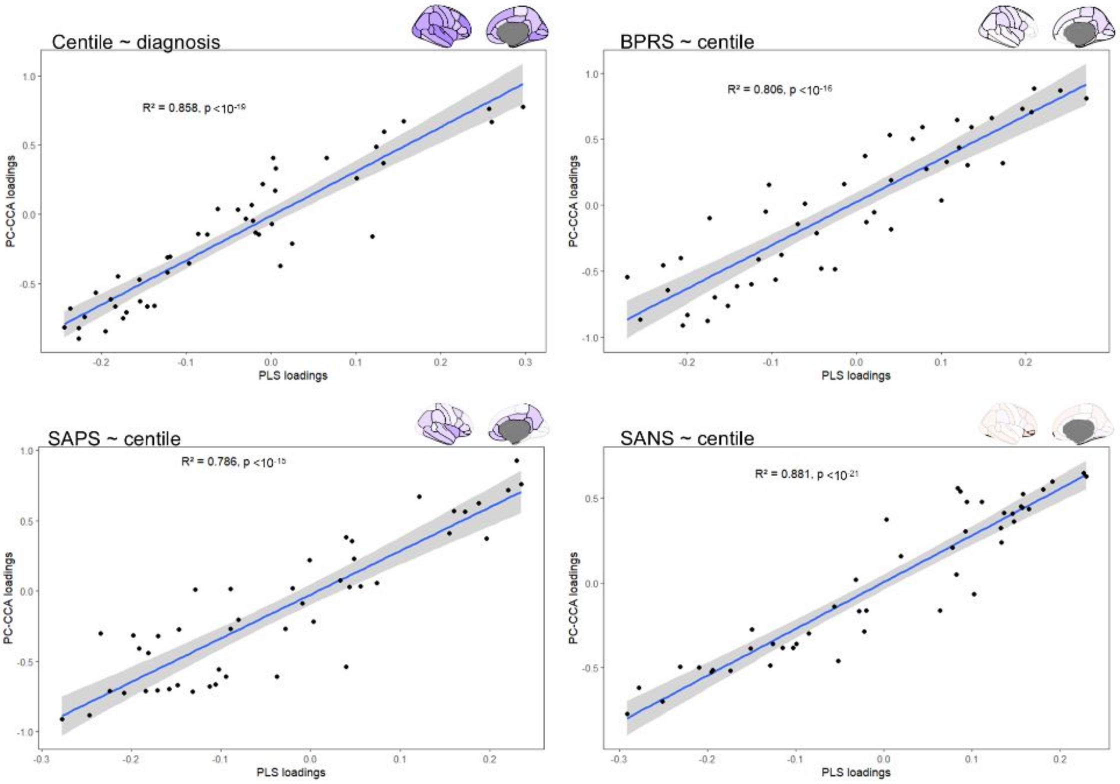

Fig. S12. **Correlation between PCA-CCA and PLS loadings.** Scatterplots showing the similarity of PCA-CCA and PLS loadings derived from mapping neurobiological features into the four significant maps of interest presented in Figs 4B and 4C.

Supplementary Tables

	Baseline	1 year	3 years	10 years
Age inclusion	30.08 ± 6.82			
Years of Education	10.12 ± 2.55			
Global Cognitive Functioning Attention	0.07 ± 1.02	0.2 ± 1.22	0.18 ± 0.7	0.29 ± 0.58
Global Cognitive Functioning average	-0.34 ± 0.65	0.24 ± 0.67	0.17 ± 0.57	0.19 ± 0.49
Global Cognitive Functioning Executive Function	-0.18 ± 1.14	0.24 ± 0.71	0.24 ± 0.66	0.04 ± 0.63
Global Cognitive Functioning Motor Dexterity	-0.31 ± 1.14	0.17 ± 0.68	0.08 ± 1.11	0.51 ± 0.74
Global Cognitive Functioning Processing Speed	-0.3 ± 0.98	0.29 ± 0.56	0.28 ± 0.79	0.38 ± 0.89
Global Cognitive Functioning Verbal Memory	-1.67 ± 1.45	-0.5 ± 1.18	-0.6 ± 1.19	-0.72 ± 1.17
Global Cognitive Functioning Visual Memory	0.09 ± 1.02	0.67 ± 0.89	0.89 ± 0.72	0.6 ± 0.65
Global Cognitive Functioning Working Memory	-0.07 ± 0.88	0.62 ± 1.04	0.12 ± 0.86	0.2 ± 1.12

Table S1. Demographic and cognitive data for male control participants (mean ± standard deviation.

	Baseline	1 year	3 years	10 years
Age inclusion	29.83 ± 8.16			
Years of Education	11.64 ± 2.64			
Global Cognitive Functioning Attention	-0.11 ± 1.12	-0.01 ± 1.22	0.29 ± 0.46	-0.1 ± 1.16
Global Cognitive Functioning average	-0.11 ± 0.55	-0.01 ± 0.6	0.15 ± 0.44	0.12 ± 0.46
Global Cognitive Functioning Executive Function	0.18 ± 0.76	0.36 ± 0.59	0.21 ± 0.49	0.12 ± 0.59
Global Cognitive Functioning Motor Dexterity	0.28 ± 0.8	0.47 ± 0.61	0.8 ± 0.53	0.5 ± 0.96
Global Cognitive Functioning Processing Speed	0.33 ± 1.03	0.59 ± 0.85	0.52 ± 1.13	0.87 ± 0.77
Global Cognitive Functioning Verbal Memory	-0.89 ± 1.05	-1.08 ± 1.46	-0.79 ± 0.89	-0.61 ± 0.93
Global Cognitive Functioning Visual Memory	-0.32 ± 1.01	-0.19 ± 1.04	0.16 ± 0.53	0.04 ± 0.69
Global Cognitive Functioning Working Memory	-0.21 ± 0.94	-0.22 ± 0.65	-0.15 ± 0.86	-0.02 ± 0.79

Table S2. Demographic and cognitive data for female control participants (mean ± standard deviation.

	Baseline	1 year	3 years	10 years
Age inclusion	27.62 ± 7.49			
Age of onset	26.86 ± 7.5			
Years of Education	9.59 ± 3.03			
BPRS depression	2.4 ± 0.89	1.27 ± 0.6	1.26 ± 0.49	1.21 ± 0.38
BPRS Disorientation	3.16 ± 1.74	1.95 ± 1.3	1.72 ± 1.23	1.75 ± 0.96
BPRS Mania	2.56 ± 0.94	1.31 ± 0.51	1.26 ± 0.53	1.15 ± 0.27
BPRS Negative	1.79 ± 0.85	1.37 ± 0.52	1.38 ± 0.73	1.24 ± 0.41
BPRS Positive	3.33 ± 0.72	1.42 ± 0.7	1.23 ± 0.51	1.2 ± 0.4
BPRS Total	64.15 ± 14.06	33.55 ± 12.12	31.45 ± 11.56	29.81 ± 7.33
Global Cognitive Functioning Attention	-1.86 ± 3.67	-1.75 ± 4.63	-1.3 ± 3.29	-1 ± 2.41
Global Cognitive Functioning average	-1.33 ± 1.15	-0.98 ± 1.41	-0.95 ± 0.95	-1.03 ± 1.15
Global Cognitive Functioning Executive Function	-1.07 ± 1.86	-0.64 ± 1.45	-0.76 ± 1.52	-1.02 ± 2.32
Global Cognitive Functioning Motor Dexterity	-1.27 ± 2.6	-0.94 ± 3.74	-0.67 ± 1.41	-1.12 ± 2.25
Global Cognitive Functioning Processing Speed	-1.69 ± 1.06	-1.17 ± 1.3	-1.25 ± 1.08	-0.64 ± 1
Global Cognitive Functioning Verbal Memory	-2.5 ± 1.32	-2.19 ± 1.5	-2.22 ± 1.4	-2.33 ± 1.29
Global Cognitive Functioning Visual Memory	-0.44 ± 1	0.09 ± 1.05	-0.13 ± 0.99	-0.6 ± 0.84
Global Cognitive Functioning Working Memory	-0.43 ± 0.78	-0.5 ± 0.8	-0.32 ± 0.78	-0.47 ± 0.87
SANS Global Rating of Affective Flattening	1.24 ± 1.49	1.3 ± 1.48	0.95 ± 1.39	1.13 ± 1.24
SANS Global Rating of Alogia	0.84 ± 1.46	0.67 ± 1.13	0.78 ± 1.28	0.72 ± 1.12
SANS Global Rating of Anhedonia Asociality	1.53 ± 1.8	1.48 ± 1.68	1.14 ± 1.71	1.24 ± 1.67
SANS Global Rating of Attention	1.85 ± 1.96	0.45 ± 1.12	0.57 ± 1.28	0.17 ± 0.5
SANS Global Rating of Avolition Apathy	1.07 ± 1.69	1.39 ± 1.7	1.09 ± 1.61	0.81 ± 1.37
SANS Total	1.17 ± 1.31	1.21 ± 1.3	0.99 ± 1.34	0.98 ± 1.18
SAPS Global rating of bizarre behaviour	4.18 ± 1.42	0.45 ± 1.09	0.33 ± 0.99	0.15 ± 0.6
SAPS Global rating of delusions	4.87 ± 0.52	1.09 ± 1.62	0.66 ± 1.3	0.69 ± 1.15
SAPS Global rating of hallucinations	2.65 ± 2.32	0.31 ± 1.02	0.37 ± 1.09	0.22 ± 0.77
SAPS Global rating of positive formal thought disorder	0.99 ± 1.7	0.09 ± 0.54	0.13 ± 0.59	0.06 ± 0.3
SAPS Total	3.17 ± 0.82	0.49 ± 0.77	0.37 ± 0.79	0.28 ± 0.54

Table S3. Demographic, cognitive and clinical data for male FEP participants (mean ± standard deviation.

	Baseline	1 year	3 years	10 years
Age inclusion	32.94 ± 9.61			
Age of onset	32.13 ± 9.52			
Years of Education	11.26 ± 3.42			
BPRS Depression	2.5 ± 0.95	1.31 ± 0.4	1.21 ± 0.3	1.48 ± 0.61
BPRS Disorientation	2.7 ± 1.73	1.34 ± 0.65	1.29 ± 0.59	1.5 ± 0.61
BPRS Mania	2.6 ± 1.1	1.13 ± 0.26	1.12 ± 0.19	1.17 ± 0.28
BPRS Negative	1.83 ± 0.87	1.19 ± 0.37	1.18 ± 0.35	1.21 ± 0.48
BPRS Positive	3.32 ± 0.79	1.06 ± 0.24	1.1 ± 0.27	1.15 ± 0.33
BPRS Total	64.07 ± 15.65	28.13 ± 5.31	27.77 ± 5.3	30.27 ± 6.99
Global Cognitive Functioning Attention	-2.39 ± 3.88	-2.36 ± 4.24	-1.72 ± 3.59	-1.87 ± 4.08
Global Cognitive Functioning average	-1.27 ± 1.15	-0.88 ± 1.33	-0.77 ± 1.03	-0.9 ± 1.1
Global Cognitive Functioning Executive Function	-1.22 ± 2.08	-0.91 ± 2.72	-0.68 ± 1.68	-0.6 ± 1.37
Global Cognitive Functioning Motor Dexterity	-0.88 ± 1.91	-0.08 ± 1.19	-0.32 ± 1.23	-0.62 ± 1.4
Global Cognitive Functioning Processing Speed	-0.99 ± 1.07	-0.43 ± 1.13	-0.44 ± 1.27	-0.41 ± 0.95
Global Cognitive Functioning Verbal Memory	-2.11 ± 1.44	-1.94 ± 1.36	-1.46 ± 1.4	-1.61 ± 1.37
Global Cognitive Functioning Visual Memory	-0.69 ± 0.94	-0.21 ± 1.25	-0.49 ± 0.81	-0.61 ± 0.86
Global Cognitive Functioning Working Memory	-0.55 ± 1.02	-0.21 ± 0.94	-0.27 ± 0.78	-0.56 ± 0.85
SANS Global Rating of Affective Flattening	0.79 ± 1.32	0.71 ± 1.14	0.45 ± 0.89	0.81 ± 1.29
SANS Global Rating of Alogia	0.63 ± 1.31	0.39 ± 1.03	0.26 ± 0.68	0.54 ± 1.07
SANS Global Rating of Anhedonia Asociality	0.98 ± 1.6	1.05 ± 1.54	0.66 ± 1.09	1.43 ± 1.66
SANS Global Rating of Attention	2.06 ± 1.92	0.45 ± 1.16	0.13 ± 0.34	0.41 ± 0.83
SANS Global Rating of Avolition Apathy	0.68 ± 1.38	0.74 ± 1.11	0.49 ± 0.93	0.76 ± 1.12
SANS Total	0.77 ± 1.14	0.72 ± 1.02	0.47 ± 0.74	0.89 ± 1.06
SAPS Global rating of bizarre behaviour	4.2 ± 1.29	0.05 ± 0.32	0.19 ± 0.62	0.14 ± 0.54
SAPS Global rating of delusions	4.75 ± 0.75	0.18 ± 0.8	0.4 ± 1.12	0.51 ± 1.22
SAPS Global rating of hallucinations	2.27 ± 2.29	0.11 ± 0.51	0.06 ± 0.3	0.19 ± 0.74
SAPS Global rating of positive formal thought disorder	1.25 ± 1.8	0 ± 0	0.02 ± 0.14	0.16 ± 0.69
SAPS Total	3.12 ± 0.92	0.09 ± 0.31	0.17 ± 0.41	0.25 ± 0.62

Table S4. Demographic, cognitive and clinical data for female FEP participants (mean ± standard deviation.

DK area	Mesulam Area
inferiorparietal	heteromodal
middletemporal	heteromodal
parsorbitalis	heteromodal
parstriangularis	heteromodal
precuneus	heteromodal
rostralmiddlefrontal	heteromodal
superiorfrontal	heteromodal
superiorparietal	heteromodal
supramarginal	heteromodal
frontalpole	heteromodal
cuneus	idiotypic
lingual	idiotypic
paracentral	idiotypic
pericalcarine	idiotypic
postcentral	idiotypic
precentral	idiotypic
caudalanteriorcingulate	paralimbic
entorhinal	paralimbic
isthmuscingulate	paralimbic
lateralorbitofrontal	paralimbic
medialorbitofrontal	paralimbic
parahippocampal	paralimbic
posteriorcingulate	paralimbic
rostralanteriorcingulate	paralimbic
temporalpole	paralimbic
insula	paralimbic
bankssts	unimodal
caudalmiddlefrontal	unimodal
fusiform	unimodal
inferiortemporal	unimodal
lateraloccipital	unimodal
parsopercularis	unimodal
superiortemporal	unimodal
transversetemporal	unimodal

Table S5. Spatial overlap between Desikan-Killiany and Mesulam zones.

Abbreviation	Neurobiological feature	Type

5-HT_{1A}	Serotonin receptor	Neurotransmitter
5-$HT1_B$	Serotonin receptor	Neurotransmitter
5-HT_{2A}	Serotonin receptor	Neurotransmitter
5-HT_4	Serotonin receptor	Neurotransmitter
5-HT_6	Serotonin receptor	Neurotransmitter
5-HTT	Serotonin transporter	Neurotransmitter
H_3	Histamine receptor	Neurotransmitter
D_1	Dopamine receptor	Neurotransmitter
D_2	Dopamine receptor	Neurotransmitter
DAT	Dopamine transporter	Neurotransmitter
NET	Norepinephrine transporter	Neurotransmitter
$\alpha_4\beta_2$	Acetylcholine receptor	Neurotransmitter
M_1	Acetylcholine receptor	Neurotransmitter
VAChT	Acetylcholine transporter	Neurotransmitter
CB_1	Cannabinoid receptor	Neurotransmitter
MOR	Opioid receptor	Neurotransmitter
$mGluR_5$	Glutamate receptor	Neurotransmitter
NMDA	Glutamate receptor	Neurotransmitter
GABA	GABA receptor	Neurotransmitter
Astro	Astrocytes	Cell type
Endo	Endothelial cells	Cell type
Micro	Microglia	Cell type
Oligo	Oligodendrocytes	Cell type
OPC	Oligodendrocytes precursors	Cell type
Neuro-Ex	Excitatory neurons	Cell type
Neuro-In	Inhibitory neurons	Cell type
Layer I	Layer I	Layer thickness
Layer II	Layer II	Layer thickness
Layer III	Layer III	Layer thickness
Layer IV	Layer IV	Layer thickness
Layer V	Layer V	Layer thickness
Layer VI	Layer VI	Layer thickness
Myelin	Myelin	Microstructure
Thickness	Cortical thickness	Microstructure
Gene PC1	Gene expression PC1	Microstructure
Neurotransmitter PC1	Neurotransmitter PC1	Microstructure
Synapse density	Synapse density	Microstructure
Evolutionary exp.	Evolutionary expansion	Cortical expansion
Developmental exp.	Developmental expansion	Cortical expansion
Scaling PNC	Allometric scaling from Philadelphia Neurodevelopmental Cohort	Cortical expansion
Scaling NIH	Allometric scaling from National Institutes of Health	Cortical expansion
CBF	Cerebral blood flow	Metabolism
CBV	Cerebral blood volume	Metabolism
$CMRO_2$	Oxygen metabolism	Metabolism
CMRGlu	Glucose metabolism	Metabolism
Glycolytic index	Glycolytic index	Metabolism

Table S6. Molecular, microarchitectural and metabolic features included in the neurobiological mapping.

		Total grey matter volume centile	Total white matter volume centile
**Intercept**	**t-value**	29.615	28.34
	**p-value**	$< 10^{-15}$	$< 10^{-15}$
**Diagnosis**	**t-value**	-6.20	-1.67
	**p-value**	$< 10^{-8}$	0.095
**Age of Inclusion**	**t-value**	4.52	3.75
	**p-value**	$<10^{-5}$	$<10^{-4}$
**Sex**	**t-value**	-2.27	3.75
	**p-value**	0.023	$<10^{-4}$
**Etiv**	**t-value**	18.03	16.47
	**p-value**	$< 10^{-15}$	$< 10^{-15}$

Table S7. Linear multiple regression of total grey and white matter volume centiles (baseline data only). Statistically significant values are represented in red (p-value<0.05).

Model	Multiple R squared	Adjusted R squared	p-value
Baseline (GMV)	0.43	0.42	$<10^{-15}$
Baseline (WMV)	0.36	0.36	$<10^{-15}$
	R marginal	R conditional	
Longitudinal structural	0.42	0.94	
Cognition longitudinal	0.26	0.76	
SAPS longitudinal	0.93	0.95	
SANS longitudinal	0.04	0.90	
BPRS longitudinal	0.64	0.97	

Table S8. Fitting estimation for global models.

		Total grey matter volume centile	Total white matter volume centile
**Intercept**	**t-value**	29.48	28.94
	**p-value**	< 10^{-15}	<10^{-15}
**Diagnosis**	**t-value**	-4.55	-1.71
	**p-value**	<10^{-5}	0.087
**Age of Inclusion**	**t-value**	4.49	3.71
	**p-value**	<10^{-5}	<10^{-3}
**Sex**	**t-value**	-2.64	-1.94
	**p-value**	0.008	0.05
**Etiv**	**t-value**	19.28	17.35
	**p-value**	<10^{-15}	<10^{-15}
**Medication**	**t-value**	-3.31	0.57
	**p-value**	<10^{-3}	0.57
**Time**	**t-value**	-0.8	1.60
	**p-Value**	0.42	0.087
**Time: FEP**	**t-value**	4.02	-2.45
	**p-value**	<10^{-4}	0.015
**Medication : Time**	**t-value**	-5.04	-1.25
	**p-value**	<10^{-6}	0.21

Table S9. Linear mixed regression of total grey and white matter volume centiles (longitudinal data). Significant values in red (p-value <0.05).

Principal Components Analysis of cognitive subscales	PC1	PC2	PC3
**Global Cognitive Functioning Verbal Memory**	0.37	-0.4	-0.58
**Global Cognitive Functioning Visual Memory**	0.38	-0.23	0.04
**Global Cognitive Functioning Processing Speed**	0.41	0.03	-0.42
**Global Cognitive Functioning Working Memory**	0.34	-0.53	0.53
**Global Cognitive Functioning Executive Function**	0.40	0.09	0.36
**Global Cognitive Functioning Motor Dexterity**	0.37	0.54	-0.12
**Global Cognitive Functioning Attention**	0.37	0.45	0.24

Table S10. Principal component analysis of cognitive subscales data.

	Longitudinal		Cognition		BPRS		SAPS		SANS	
	β	P value	β	P value	β	P value	β	P value	β	P value
**Centile**			0.504	0.040	-0.091	0.032	0.015	0.954	-0.126	0.166
**Time**	0.014	0.121	0.133	0.004	-0.379	$<10^{-15}$	-0.188	$<10^{-4}$	-2.87	$<10^{-15}$
**Etiv**	0.183	$<10^{-15}$	0.021	0.768	-0.007	0.625	-0.056	0.530	0.020	0.428
**Age of inclusion**	0.023	0.055	-0.147	0.015	-0.011	0.404	-0.055	0.479	-0.036	0.074
**Sex (Female)**	-0.035	0.162	0.164	0.198	-0.020	0.476	-0.441	0.007	-0.058	0.183
**Highly Medicated**	-0.01	0.417	0.125	0.237	-0.250	$<10^{-15}$	0.168	0.018	0.348	0.003
**Highly Medicated: Time**	-0.023	0.014	-0.209	0.011	0.354	$<10^{-15}$	0.140	0.029	2.600	$<10^{-15}$

Table S11. LMMs after categorizing patients into low (CPZ≤300) and high medication groups (CPZ>300).

Clinical scale	Assessment	FEP cannabis users	FEP cannabis non-users	p Value
Average Cognitive Functioning	1	-1.24 ± 0.86	-1.35 ± 1.30	0.5391
	2	-0.77 ± 0.95	-0.98 ± 1.44	0.5956
	3	-0.89 ± 1.16	-0.88 ± 0.95	0.9754
	10	-0.91 ± 1.35	-0.98 ± 1.12	0.8873
SAPS	1	3.16 ± 0.89	3.14 ± 0.84	0.8131
	2	0.86 ± 0.87	0.23 ± 0.56	0.4911
	3	0.67 ± 1.24	0.24 ± 0.55	0.4808
	10	0.57 ± 0.62	0.24 ± 0.56	0.4907
SANS	1	0.99 ± 1.20	1.03 ± 1.30	0.7624
	2	1.01 ± 1.16	1.04 ± 1.25	0.9346
	3	0.97 ± 1.25	0.78 ± 1.18	0.5693
	10	0.96 ± 0.88	0.94 ± 1.15	0.9336
BPRS	1	63.55 ± 13.69	64.48 ± 15.31	0.6189
	2	35.74 ± 12.92	30.67 ± 9.73	0.4879
	3	36.61 ± 17.99	29.21 ± 7.87	0.473
	10	32.57 ± 8.12	29.79 ± 7.08	0.4995

Table S12. Mean (± standard deviation) clinical scores stratified for cannabis users and non-users. P-values were computed using the Wilcoxon signed-rank test.

Variable	Assessment transition	N dropout	N continue	Median dropout	Median continue	P Value	P Value Adj FDR
Cognition	1□2	211	91	-1.00	-1.09	0.448	0.672
	2□3	21	61	-0.25	-0.75	0.048	0.144
	3□10	73	48	-0.72	-0.74	0.745	0.745
SAPS	1□2	248	103	3.50	3.00	0.540	0.614
	2□3	26	79	0.00	0.00	0.330	0.614
	3□10	91	55	0.00	0.00	0.614	0.614
SANS	1□2	248	103	0.25	0.75	0.069	0.207
	2□3	26	79	0.50	0.50	0.562	0.772
	3□10	91	55	0.25	0.25	0.772	0.772
BPRS	1□2	248	103	64.00	60.00	0.072	0.216
	2□3	26	79	27.00	27.00	0.703	0.703
	3□10	90	55	26.00	27.00	0.590	0.703

Table S13. Comparison of clinical characteristics between participants who continued and those who dropped out at subsequent assessments. Results are presented for transitions from assessment 1 to 2, 2 to 3, and 3 to 10. The table includes median values, sample sizes, and Wilcoxon rank-sum p-values for cognition, SAPS, SANS, and BPRS. P-values were corrected for multiple testing using the False Discovery Rate (FDR) adjustment.

REFERENCES

1. Gallardo-Ruiz R, Crespo-Facorro B, Setién-Suero E, Tordesillas-Gutierrez D. Long-Term Grey Matter Changes in First Episode Psychosis: A Systematic Review. Psychiatry Investig. 2019;16:336–345.
2. Andreasen NC, Flaum M, Arndt S. The Comprehensive Assessment of Symptoms and History (CASH). An instrument for assessing diagnosis and psychopathology. Arch Gen Psychiatry. 1992;49:615–623.

cortical Area	T [illegible]	
	diagnosis	age of inclusion
bankssts	-4.59	3.24
caudalanteriorcingulate	-1.80	0.25
caudalmiddlefrontal	-3.29	0.41
cuneus	-3.56	3.11
entorhinal	1.68	0.95
frontalpole	-1.39	2.08
fusiform	-3.19	2.54
inferiorparietal	-4.23	5.15
inferiortemporal	-2.35	2.21
insula	-4.58	-1.88
isthmuscingulate	-2.77	1.74
lateraloccipital	-4.37	4.25
lateralorbitofrontal	-4.08	2.09
lingual	-4.16	3.14
medialorbitofrontal	-3.47	-0.02
middletemporal	-3.80	2.36
paracentral	-2.44	4.17
parahippocampal	-3.19	0.49
parsopercularis	-2.83	1.76
parsorbitalis	-4.28	3.12
parstriangularis	-2.16	2.59
pericalcarine	-2.45	1.27
postcentral	-4.46	4.51
posteriorcingulate	-3.35	2.79
precentral	-3.89	4.82
precuneus	-3.71	4.76
rostralanteriorcingulate	-2.78	-1.07
rostralmiddlefrontal	-5.29	4.46
superiorfrontal	-4.73	1.08
superiorparietal	-3.64	5.24
superiortemporal	-5.14	3.69
supramarginal	-4.43	2.26
temporalpole	-1.71	0.42
transversetemporal	-4.99	1.88

values		p	
sex	etiv	diagnosis	age of inclusion
-0.86	9.51	0.000	0.004
-1.45	9.95	0.080	0.828
-0.12	11.73	0.002	0.725
-1.24	7.49	0.001	0.005
-0.69	6.21	0.096	0.399
-2.55	4.18	0.166	0.062
-1.80	11.33	0.002	0.024
-1.92	11.48	0.000	0.000
-2.16	11.68	0.022	0.050
-0.34	11.35	0.000	0.090
-0.44	11.09	0.008	0.112
-2.97	8.69	0.000	0.000
-1.96	12.30	0.000	0.062
-1.15	7.56	0.000	0.005
-0.44	12.04	0.001	0.981
-2.06	10.79	0.000	0.037
-1.76	10.88	0.018	0.000
0.08	8.06	0.002	0.706
-0.52	8.25	0.007	0.111
-0.84	6.92	0.000	0.005
-1.81	6.08	0.035	0.022
-1.37	5.44	0.018	0.266
-1.58	11.25	0.000	0.000
-0.36	12.81	0.001	0.013
-2.58	10.20	0.000	0.000
-3.65	12.54	0.000	0.000
0.22	10.79	0.008	0.349
-0.39	13.88	0.000	0.000
-1.33	13.15	0.000	0.349
-4.38	11.61	0.001	0.000
-1.53	11.85	0.000	0.001
-1.73	12.06	0.000	0.045
-5.02	5.58	0.093	0.725
0.51	7.22	0.000	0.090

values	
sex	etiv
0.597	0.000
0.298	0.000
0.929	0.000
0.369	0.000
0.697	0.000
0.063	0.000
0.204	0.000
0.189	0.000
0.150	0.000
0.806	0.000
0.804	0.000
0.027	0.000
0.189	0.000
0.403	0.000
0.804	0.000
0.170	0.000
0.204	0.000
0.937	0.000
0.798	0.000
0.597	0.000
0.204	0.000
0.326	0.000
0.260	0.000
0.806	0.000
0.063	0.000
0.003	0.000
0.880	0.000
0.806	0.000
0.329	0.000
0.000	0.000
0.271	0.000
0.204	0.000
0.000	0.000
0.798	0.000

	T va				
cortical area	diagnosis	time	etiv	age inclusio	sex female
bankssts	-3.75	-0.98	10.07	3.33	-0.93
insula	-1.42	-3.20	9.78	0.42	-1.40
isthmuscingulate	-2.17	-5.73	12.31	0.59	-0.37
medialorbitofrontal	-3.25	5.16	7.61	2.69	-1.25
precuneus	1.88	-3.41	7.10	1.08	-0.87
transversetemporal	-0.39	-5.39	4.84	2.26	-2.72
caudalmiddlefrontal	-2.29	-0.56	11.88	2.44	-2.07
fusiform	-3.23	2.58	12.23	5.01	-2.02
parahippocampal	-1.52	-4.85	12.31	2.33	-2.35
superiorfrontal	-3.43	-9.97	11.95	-1.87	-0.13
parsopercularis	-1.73	-0.80	11.52	1.52	-0.48
supramarginal	-4.23	5.60	9.16	4.18	-3.16
superiorparietal	-3.25	-3.25	13.28	2.13	-2.26
inferiortemporal	-3.69	3.60	8.09	2.64	-1.31
posteriorcingulate	-1.81	-7.71	13.00	0.00	-0.69
rostralanteriorcingulate	-3.02	-1.64	11.42	2.45	-2.30
inferiorparietal	-0.86	2.24	12.04	3.98	-1.66
caudalanteriorcingulate	-2.55	-2.77	8.62	0.44	-0.05
middletemporal	-2.04	-0.61	8.93	1.63	-0.37
paracentral	-3.66	-3.42	7.84	3.19	-0.96
rostralmiddlefrontal	-1.72	0.63	6.66	2.60	-2.14
precentral	-2.37	4.72	5.63	0.92	-1.38
superiortemporal	-3.37	3.97	11.78	4.53	-1.94
lateralorbitofrontal	-2.52	-3.55	12.94	2.72	-0.69
postcentral	-2.90	4.36	11.20	4.90	-2.89
frontalpole	-2.35	4.15	13.52	4.51	-3.67
lingual	-2.30	-2.67	11.45	-1.38	0.15
parsorbitalis	-4.49	-2.95	14.70	4.52	-0.64
temporalpole	-3.24	-3.75	14.22	1.00	-1.64
cuneus	-2.14	4.03	12.33	5.02	-4.51
pericalcarine	-4.35	1.60	12.77	3.55	-1.97
parstriangularis	-3.55	-0.73	12.72	2.31	-1.81
entorhinal	-0.93	-4.75	6.69	0.18	-4.79
lateraloccipital	-4.13	0.38	7.95	1.59	0.38

lues				
medication	diagnosis : time	tiempo : medication	diagnosis	time
0.32	4.69	-5.21	0.001	0.396
-2.94	2.15	-2.64	0.170	0.002
-2.12	3.84	-3.97	0.045	0.000
-0.88	0.78	-3.02	0.003	0.000
-2.17	0.31	-1.35	0.080	0.001
-2.85	1.41	-3.71	0.697	0.000
-3.68	3.33	-4.64	0.035	0.591
-3.42	3.02	-3.36	0.003	0.014
-2.89	2.97	-3.89	0.145	0.000
-4.43	4.79	-4.15	0.002	0.000
-3.03	4.37	-2.54	0.102	0.499
-3.46	0.17	-2.15	0.000	0.000
-3.29	1.57	-2.74	0.003	0.002
-2.68	1.25	-2.90	0.001	0.001
-2.35	4.28	-3.41	0.088	0.000
-3.42	2.36	-3.37	0.006	0.132
-1.13	2.45	-2.01	0.402	0.035
-3.38	3.79	-4.21	0.022	0.009
-4.06	3.01	-2.06	0.057	0.574
-2.60	1.48	-2.62	0.001	0.001
-3.45	0.93	-2.03	0.102	0.574
-1.11	-0.21	-0.84	0.032	0.000
-1.01	1.91	-2.20	0.003	0.000
-1.50	2.72	-2.09	0.023	0.001
-3.34	2.44	-1.50	0.008	0.000
-2.97	3.98	-3.39	0.032	0.000
-3.87	2.46	-4.91	0.035	0.011
-2.44	2.83	-3.79	0.000	0.005
-3.28	3.87	-4.62	0.003	0.000
-1.59	2.93	-1.77	0.046	0.000
-2.05	1.87	-3.86	0.000	0.138
-3.05	3.80	-3.97	0.002	0.529
-2.52	0.84	-1.54	0.377	0.000
-0.57	3.89	-6.36	0.000	0.703

p values				
etiv	age inclusion	sex female	medication	diagnosis : time
0.000	0.003	0.525	0.750	0.000
0.000	0.720	0.303	0.007	0.049
0.000	0.628	0.780	0.047	0.001
0.000	0.019	0.343	0.401	0.475
0.000	0.351	0.547	0.043	0.805
0.000	0.041	0.038	0.009	0.199
0.000	0.030	0.121	0.002	0.003
0.000	0.000	0.125	0.003	0.007
0.000	0.038	0.092	0.008	0.007
0.000	0.097	0.924	0.000	0.000
0.000	0.176	0.769	0.006	0.000
0.000	0.000	0.014	0.003	0.867
0.000	0.055	0.093	0.003	0.158
0.000	0.021	0.326	0.013	0.258
0.000	0.996	0.641	0.028	0.000
0.000	0.030	0.092	0.003	0.030
0.000	0.000	0.205	0.305	0.025
0.000	0.720	0.957	0.003	0.001
0.000	0.153	0.780	0.001	0.007
0.000	0.005	0.522	0.016	0.184
0.000	0.022	0.112	0.003	0.411
0.000	0.423	0.303	0.305	0.856
0.000	0.000	0.129	0.342	0.083
0.000	0.019	0.641	0.162	0.014
0.000	0.000	0.027	0.003	0.025
0.000	0.000	0.003	0.007	0.001
0.000	0.220	0.924	0.001	0.025
0.000	0.000	0.655	0.023	0.010
0.000	0.385	0.205	0.003	0.001
0.000	0.000	0.000	0.142	0.008
0.000	0.002	0.129	0.053	0.088
0.000	0.038	0.159	0.006	0.001
0.000	0.881	0.000	0.019	0.453
0.000	0.160	0.780	0.588	0.001

tiempo : medication
0.000
0.014
0.000
0.005
0.182
0.001
0.000
0.002
0.000
0.000
0.017
0.043
0.011
0.007
0.002
0.002
0.053
0.000
0.050
0.014
0.052
0.399
0.040
0.048
0.144
0.002
0.000
0.000
0.000
0.088
0.000
0.000
0.136
0.000

cortical area			
	time	diagnosis	etiv
bankssts	1.52	-7.21	1.74
audalanteriorcingulat	1.18	-7.13	1.96
caudalmiddlefrontal	1.59	-6.49	2.08
cuneus	2.27	-7.25	2.11
entorhinal	2.10	-5.84	2.57
frontalpole	1.12	-6.95	2.08
fusiform	2.54	-8.02	0.90
inferiorparietal	2.96	-6.66	1.38
inferiortemporal	2.34	-6.80	1.51
insula	0.99	-7.90	1.03
isthmuscingulate	1.64	-7.61	1.92
lateraloccipital	2.08	-7.18	1.61
lateralorbitofrontal	1.94	-8.29	0.79
lingual	2.08	-7.09	2.22
medialorbitofrontal	1.82	-7.84	1.33
middletemporal	1.74	-7.01	1.65
paracentral	2.36	-7.29	1.78
parahippocampal	1.84	-6.96	2.00
parsopercularis	0.78	-6.47	2.01
parsorbitalis	1.07	-7.17	1.83
parstriangularis	1.46	-7.17	1.86
pericalcarine	2.22	-6.37	2.65
postcentral	1.52	-7.66	1.67
posteriorcingulate	1.19	-6.95	1.40
precentral	2.05	-6.92	1.71
precuneus	2.87	-7.72	1.03
ostralanteriorcingulat	1.84	-7.10	1.58
rostralmiddlefrontal	2.70	-7.61	1.39
superiorfrontal	2.58	-7.58	1.61
superiorparietal	2.81	-8.41	0.73
superiortemporal	1.19	-7.26	1.49
supramarginal	2.18	-7.72	1.90
temporalpole	1.13	-7.18	1.79
transversetemporal	1.76	-7.23	1.91

t values				
age of inclusion	sex female	medication	centile	diagnosis :time
-4.15	1.67	2.83	-0.53	1.43
-4.11	1.81	2.91	-0.62	1.51
-4.13	1.78	2.85	-0.28	1.50
-4.11	1.78	2.83	-0.89	1.24
-4.12	1.77	2.78	-0.75	1.83
-4.21	1.83	2.91	-0.18	1.60
-4.10	2.06	2.93	-0.55	1.42
-4.42	1.91	2.77	0.27	1.70
-4.18	1.90	2.86	-0.10	1.72
-4.12	1.80	3.07	-0.47	1.67
-4.10	1.76	2.86	-1.08	1.45
-4.38	1.97	2.93	0.00	1.16
-4.28	1.91	3.09	-0.61	1.45
-4.06	1.76	2.78	-1.11	1.36
-4.21	1.80	2.99	-0.62	1.60
-4.17	1.89	2.93	-0.08	1.43
-4.20	1.70	2.81	-0.79	1.26
-4.16	1.82	2.83	-0.27	1.58
-4.12	1.77	2.90	-0.12	1.53
-4.21	1.81	2.92	-0.14	1.55
-4.32	1.88	2.95	-0.21	1.43
-4.07	1.70	2.76	-1.22	1.53
-4.17	1.76	2.85	-0.68	1.25
-4.34	1.82	2.90	0.08	1.45
-4.21	1.76	2.84	-0.17	1.27
-4.29	1.95	2.84	-0.43	1.19
-4.07	1.71	2.89	-0.37	1.52
-4.32	1.84	2.95	-0.63	1.24
-4.24	1.81	2.91	-0.81	1.32
-4.49	2.23	2.75	-0.33	0.99
-4.16	1.82	2.90	-0.23	1.36
-4.03	1.81	2.82	-1.31	1.38
-4.13	2.07	2.96	-0.01	1.62
-4.22	1.76	2.82	-0.26	1.21

diagnosis : centile	tiempo : medicacion	time : centile
1.51	-5.05	-0.66
1.27	-4.95	-0.16
0.38	-5.12	-0.83
1.16	-5.28	-1.62
0.04	-5.31	-1.58
0.80	-5.01	-0.14
2.46	-5.19	-2.00
0.86	-5.43	-2.71
1.00	-5.32	-1.81
2.29	-4.99	0.04
1.71	-5.10	-0.81
1.05	-5.17	-1.49
2.75	-5.11	-1.24
1.14	-5.23	-1.39
1.95	-5.13	-1.17
0.99	-5.14	-0.99
1.47	-5.08	-1.76
0.70	-5.22	-1.11
0.55	-5.03	0.29
1.24	-5.12	-0.18
1.21	-5.13	-0.65
0.19	-5.36	-1.56
1.65	-5.05	-0.61
1.14	-4.99	-0.19
0.96	-5.13	-1.42
2.08	-5.04	-2.45
1.25	-5.10	-1.10
1.69	-5.25	-2.29
1.56	-5.21	-2.15
2.75	-4.99	-2.34
1.25	-5.02	-0.25
1.80	-5.20	-1.56
1.22	-5.00	-0.05
1.09	-4.98	-1.02

time	diagnosis	etiv	age of inclusion	sex female
0.176	0.000	0.165	0.000	0.096
0.281	0.000	0.162	0.000	0.090
0.166	0.000	0.162	0.000	0.090
0.090	0.000	0.162	0.000	0.090
0.093	0.000	0.162	0.000	0.090
0.290	0.000	0.162	0.000	0.090
0.064	0.000	0.392	0.000	0.090
0.058	0.000	0.206	0.000	0.090
0.084	0.000	0.185	0.000	0.090
0.332	0.000	0.335	0.000	0.090
0.158	0.000	0.162	0.000	0.090
0.093	0.000	0.168	0.000	0.090
0.112	0.000	0.443	0.000	0.090
0.093	0.000	0.162	0.000	0.090
0.125	0.000	0.216	0.000	0.090
0.134	0.000	0.168	0.000	0.090
0.084	0.000	0.162	0.000	0.092
0.125	0.000	0.162	0.000	0.090
0.436	0.000	0.162	0.000	0.090
0.301	0.000	0.162	0.000	0.090
0.188	0.000	0.162	0.000	0.090
0.091	0.000	0.162	0.000	0.092
0.176	0.000	0.168	0.000	0.090
0.281	0.000	0.206	0.000	0.090
0.093	0.000	0.165	0.000	0.090
0.058	0.000	0.335	0.000	0.090
0.125	0.000	0.168	0.000	0.092
0.061	0.000	0.206	0.000	0.090
0.064	0.000	0.168	0.000	0.090
0.058	0.000	0.468	0.000	0.090
0.281	0.000	0.185	0.000	0.090
0.091	0.000	0.162	0.000	0.090
0.290	0.000	0.162	0.000	0.090
0.134	0.000	0.162	0.000	0.090

p values		
medication	centile	diagnosis :time
0.006	0.997	0.246
0.006	0.997	0.246
0.006	0.997	0.246
0.006	0.997	0.246
0.006	0.997	0.246
0.006	0.997	0.246
0.006	0.997	0.246
0.006	0.997	0.246
0.006	0.997	0.246
0.006	0.997	0.246
0.006	0.997	0.246
0.006	0.997	0.253
0.006	0.997	0.246
0.006	0.997	0.246
0.006	0.997	0.246
0.006	0.997	0.246
0.006	0.997	0.246
0.006	0.997	0.246
0.006	0.997	0.246
0.006	0.997	0.246
0.006	0.997	0.246
0.006	0.997	0.246
0.006	0.997	0.246
0.006	0.997	0.246
0.006	0.997	0.246
0.006	0.997	0.248
0.006	0.997	0.246
0.006	0.997	0.246
0.006	0.997	0.246
0.006	0.997	0.325
0.006	0.997	0.246
0.006	0.997	0.246
0.006	0.997	0.246
0.006	0.997	0.248

diagnosis : centile	tiempo : medicacion	time : centile
0.370	0.000	0.701
0.394	0.000	0.942
0.749	0.000	0.615
0.394	0.000	0.340
0.968	0.000	0.340
0.494	0.000	0.942
0.161	0.000	0.262
0.475	0.000	0.191
0.419	0.000	0.338
0.192	0.000	0.968
0.336	0.000	0.615
0.419	0.000	0.355
0.105	0.000	0.456
0.394	0.000	0.378
0.295	0.000	0.483
0.419	0.000	0.525
0.371	0.000	0.338
0.552	0.000	0.483
0.641	0.000	0.942
0.394	0.000	0.942
0.394	0.000	0.701
0.872	0.000	0.340
0.336	0.000	0.705
0.394	0.000	0.942
0.427	0.000	0.378
0.257	0.000	0.191
0.394	0.000	0.483
0.336	0.000	0.191
0.367	0.000	0.218
0.105	0.000	0.191
0.394	0.000	0.942
0.336	0.000	0.340
0.394	0.000	0.968
0.411	0.000	0.525

cortical area	time	age inclusion	sex
bankssts	-8.94	-2.09	-1.28
caudalanteriorcingulate	-6.58	-2.39	-1.30
caudalmiddlefrontal	-6.12	-2.29	-1.28
cuneus	-9.62	-2.30	-1.32
entorhinal	-5.43	-2.24	-1.32
frontalpole	-13.60	-2.42	-1.31
fusiform	-14.16	-2.64	-1.51
inferiorparietal	-6.47	-2.25	-1.35
inferiortemporal	-6.88	-2.38	-1.31
insula	-7.18	-2.45	-1.13
isthmuscingulate	-4.13	-2.35	-1.31
lateraloccipital	-9.40	-2.37	-1.24
lateralorbitofrontal	-6.21	-2.28	-1.20
lingual	-10.80	-2.31	-1.31
medialorbitofrontal	-7.36	-2.28	-1.18
middletemporal	-7.65	-2.21	-1.45
paracentral	-7.89	-2.06	-1.44
parahippocampal	-11.09	-2.43	-1.27
parsopercularis	-8.93	-2.35	-1.23
parsorbitalis	-7.48	-2.35	-1.20
parstriangularis	-7.83	-2.49	-1.10
pericalcarine	-9.18	-2.30	-1.28
postcentral	-8.34	-2.19	-1.33
posteriorcingulate	-6.10	-2.29	-1.20
precentral	-7.92	-2.24	-1.43
precuneus	-5.11	-2.02	-1.44
rostralanteriorcingulate	-6.42	-2.46	-1.23
rostralmiddlefrontal	-9.16	-2.38	-1.22
superiorfrontal	-10.23	-2.26	-1.40
superiorparietal	-10.95	-2.13	-1.63
superiortemporal	-8.75	-2.15	-1.26
supramarginal	-9.73	-2.29	-1.50
temporalpole	-9.49	-2.25	-1.28
transversetemporal	-10.96	-2.23	-1.20

T Values				
etiv	cpz	centile	time:medication	time:centile
0.43	-10.55	-0.67	20.06	-1.55
0.24	-8.78	-3.47	17.21	-4.02
-0.13	-11.71	-2.83	21.87	-5.13
0.30	-7.68	-0.65	15.64	-0.94
0.26	-8.43	-5.66	21.20	-6.79
0.13	-1.96	3.06	22.25	4.86
1.83	-0.67	-0.77	21.87	2.99
0.09	-11.26	-3.61	21.20	-5.19
0.10	-11.89	-3.94	20.24	-5.60
0.57	-15.05	-7.81	19.80	-9.82
0.40	-13.42	-5.70	21.47	-7.91
-0.11	-8.53	-1.52	18.75	-2.32
-0.04	-13.23	-5.11	21.84	-7.23
0.34	-6.18	0.36	20.06	0.63
-0.32	-10.25	-4.62	20.77	-6.20
0.77	-13.43	-4.62	20.06	-5.66
0.90	-6.69	-1.21	21.34	-0.89
0.24	-6.06	-0.61	20.91	-0.29
0.38	-10.83	-2.66	18.02	-3.47
-0.15	-8.37	-2.99	19.97	-3.89
-0.06	-12.08	-2.74	20.39	-4.28
-0.02	-8.24	1.89	16.97	0.66
0.76	-6.14	-2.62	16.43	-2.02
0.11	-13.67	-4.26	19.99	-6.20
0.27	-9.09	-1.47	21.41	-2.23
0.93	-11.60	-4.44	21.32	-5.54
0.24	-11.15	-4.61	20.64	-5.93
-0.57	-12.07	-1.46	21.12	-3.68
0.76	-9.33	-1.14	19.77	-1.33
1.37	-2.97	-0.31	17.80	1.63
0.90	-13.43	-3.80	20.85	-4.82
1.56	-7.12	-2.84	21.23	-1.83
0.00	-10.48	-1.43	18.42	-2.57
0.49	-9.22	0.94	19.56	0.71

centile:medication	time	age inclusion	sex	etiv
6.07	0.0000	0.0386	0.2531	0.9979
4.39	0.0000	0.0325	0.2531	0.9979
8.27	0.0000	0.0325	0.2531	0.9979
2.14	0.0000	0.0325	0.2531	0.9979
4.66	0.0000	0.0325	0.2531	0.9979
-6.23	0.0000	0.0325	0.2531	0.9979
-7.85	0.0000	0.0325	0.2531	0.9979
6.91	0.0000	0.0325	0.2531	0.9979
7.70	0.0000	0.0325	0.2531	0.9979
12.11	0.0000	0.0325	0.2683	0.9979
10.33	0.0000	0.0325	0.2531	0.9979
2.81	0.0000	0.0325	0.2531	0.9979
9.64	0.0000	0.0325	0.2531	0.9979
0.05	0.0000	0.0325	0.2531	0.9979
5.59	0.0000	0.0325	0.2531	0.9979
9.45	0.0000	0.0332	0.2531	0.9979
1.94	0.0000	0.0409	0.2531	0.9979
-0.46	0.0000	0.0325	0.2531	0.9979
5.00	0.0000	0.0325	0.2531	0.9979
3.50	0.0000	0.0325	0.2531	0.9979
6.97	0.0000	0.0325	0.2693	0.9979
3.87	0.0000	0.0325	0.2531	0.9979
0.56	0.0000	0.0332	0.2531	0.9979
9.62	0.0000	0.0325	0.2531	0.9979
4.31	0.0000	0.0325	0.2531	0.9979
7.94	0.0000	0.0436	0.2531	0.9979
6.98	0.0000	0.0325	0.2531	0.9979
7.55	0.0000	0.0325	0.2531	0.9979
3.45	0.0000	0.0325	0.2531	0.9979
-3.45	0.0000	0.0361	0.2531	0.9979
9.33	0.0000	0.0358	0.2531	0.9979
1.21	0.0000	0.0325	0.2531	0.9979
4.83	0.0000	0.0325	0.2531	0.9979
4.00	0.0000	0.0325	0.2531	0.9979

p Values			
cpz	centile	time:medication	time:centile
0.0000	0.5667	0.0000	0.1511
0.0000	0.0014	0.0000	0.0001
0.0000	0.0092	0.0000	0.0000
0.0000	0.5667	0.0000	0.4081
0.0000	0.0000	0.0000	0.0000
0.0519	0.0054	0.0000	0.0000
0.5047	0.5148	0.0000	0.0048
0.0000	0.0009	0.0000	0.0000
0.0000	0.0003	0.0000	0.0000
0.0000	0.0000	0.0000	0.0000
0.0000	0.0000	0.0000	0.0000
0.0000	0.1982	0.0000	0.0318
0.0000	0.0000	0.0000	0.0000
0.0000	0.7370	0.0000	0.5462
0.0000	0.0000	0.0000	0.0000
0.0000	0.0000	0.0000	0.0000
0.0000	0.2957	0.0000	0.4224
0.0000	0.5787	0.0000	0.7699
0.0000	0.0141	0.0000	0.0009
0.0000	0.0062	0.0000	0.0002
0.0000	0.0116	0.0000	0.0000
0.0000	0.0956	0.0000	0.5428
0.0000	0.0149	0.0000	0.0620
0.0000	0.0001	0.0000	0.0000
0.0000	0.2037	0.0000	0.0383
0.0000	0.0000	0.0000	0.0000
0.0000	0.0000	0.0000	0.0000
0.0000	0.2037	0.0000	0.0004
0.0000	0.3186	0.0000	0.2230
0.0031	0.7592	0.0000	0.1348
0.0000	0.0004	0.0000	0.0000
0.0000	0.0092	0.0000	0.0912
0.0000	0.2094	0.0000	0.0165
0.0000	0.4195	0.0000	0.5245

centile:medication
0.0000
0.0000
0.0000
0.0381
0.0000
0.0000
0.0000
0.0000
0.0000
0.0000
0.0000
0.0061
0.0000
0.9612
0.0000
0.0000
0.0589
0.6637
0.0000
0.0006
0.0000
0.0002
0.6088
0.0000
0.0000
0.0000
0.0000
0.0000
0.0007
0.0007
0.0000
0.2483
0.0000
0.0001

						T
cortical area	time	age inc	sex	etiv	cpz	centile
bankssts	-3.90	-0.71	-2.68	-1.02	1.15	1.20
caudalanteriorcingulate	-1.97	-0.64	-2.59	-1.13	1.58	0.97
caudalmiddlefrontal	-4.14	-0.63	-2.67	-1.50	2.81	2.47
cuneus	-3.01	-0.68	-2.68	-0.65	1.97	0.00
entorhinal	-3.94	-0.67	-2.54	-1.19	2.51	2.45
frontalpole	-2.52	-0.98	-2.33	-1.32	3.55	3.96
fusiform	-5.77	-0.70	-2.50	-1.48	5.54	2.75
inferiorparietal	-5.41	-0.89	-2.59	-1.22	3.30	1.97
inferiortemporal	-5.76	-0.76	-2.58	-1.37	1.78	2.07
insula	-4.84	-0.68	-2.69	-0.63	1.27	0.20
isthmuscingulate	-3.76	-0.68	-2.71	-0.19	1.47	-0.88
lateraloccipital	-2.61	-0.85	-2.50	-1.05	1.91	0.95
lateralorbitofrontal	-4.59	-0.75	-2.53	-1.65	2.90	2.69
lingual	-1.46	-0.58	-2.72	-0.33	-1.63	-1.43
medialorbitofrontal	-6.03	-0.67	-2.67	-0.90	4.78	1.49
middletemporal	-4.26	-0.74	-2.51	-1.41	1.87	2.13
paracentral	-3.08	-0.67	-2.67	-0.69	1.01	0.22
parahippocampal	-5.33	-0.65	-2.70	-0.63	2.97	0.46
parsopercularis	-4.50	-0.61	-2.73	-0.11	-0.39	-1.54
parsorbitalis	-6.15	-0.96	-2.56	-1.72	5.37	4.70
parstriangularis	-4.90	-0.81	-2.57	-1.00	0.43	1.31
pericalcarine	-1.39	-0.65	-2.59	-0.96	0.78	0.89
postcentral	-6.12	-0.79	-2.60	-1.07	4.39	1.85
posteriorcingulate	-2.59	-0.69	-2.66	-0.77	-0.68	-0.01
precentral	-4.06	-0.62	-2.67	-0.53	2.45	0.07
precuneus	-2.85	-0.82	-2.51	-1.28	3.01	1.50
rostralanteriorcingulate	-4.66	-0.71	-2.65	-0.40	2.85	-0.08
rostralmiddlefrontal	-5.33	-0.90	-2.56	-1.37	2.70	2.05
superiorfrontal	-4.28	-0.72	-2.58	-1.31	2.57	1.85
superiorparietal	-5.09	-0.68	-2.60	-0.56	5.00	0.54
superiortemporal	-2.68	-0.74	-2.62	-1.11	0.77	0.91
supramarginal	-4.11	-0.68	-2.75	0.06	3.02	-1.23
temporalpole	-4.54	-0.65	-2.40	-1.09	1.23	1.79
transversetemporal	-3.88	-0.67	-2.67	-0.64	1.53	0.00

Values					
time:medication	time:centile	centile:medication	time	age inclusion	sex
3.81	1.29	0.29	0.000	0.559	0.013
3.47	-1.54	-0.29	0.052	0.559	0.013
3.65	1.23	-1.95	0.000	0.559	0.013
3.65	-0.17	-1.09	0.003	0.559	0.013
3.66	1.56	-1.78	0.000	0.559	0.013
3.99	-0.12	-2.57	0.013	0.559	0.020
5.02	3.32	-5.08	0.000	0.559	0.013
4.05	2.97	-2.47	0.000	0.559	0.013
4.18	3.50	-0.70	0.000	0.559	0.013
3.45	1.72	-0.09	0.000	0.559	0.013
3.33	1.08	-0.64	0.000	0.559	0.013
3.61	-1.03	-0.73	0.011	0.559	0.013
3.52	1.86	-2.03	0.000	0.559	0.013
3.79	-1.95	3.07	0.149	0.559	0.013
4.00	3.22	-4.42	0.000	0.559	0.013
3.38	1.21	-0.69	0.000	0.559	0.013
3.50	0.79	-0.02	0.003	0.559	0.013
3.41	2.81	-2.24	0.000	0.559	0.013
4.18	1.18	1.64	0.000	0.559	0.013
4.65	3.70	-4.87	0.000	0.559	0.013
4.11	2.13	0.81	0.000	0.559	0.013
3.38	-1.78	0.25	0.164	0.559	0.013
4.60	3.75	-3.91	0.000	0.559	0.013
4.05	-0.96	2.17	0.011	0.559	0.013
3.45	1.53	-1.58	0.000	0.559	0.013
3.96	-0.29	-2.16	0.006	0.559	0.013
3.50	1.90	-2.16	0.000	0.559	0.013
3.52	2.57	-1.73	0.000	0.559	0.013
3.24	1.21	-1.52	0.000	0.559	0.013
5.43	2.61	-4.60	0.000	0.559	0.013
3.52	-1.15	0.89	0.009	0.559	0.013
3.39	0.95	-2.31	0.000	0.559	0.013
3.55	1.76	0.03	0.000	0.559	0.017
3.31	0.43	-0.44	0.000	0.559	0.013

p Values					
etiv	cpz	centile	time:medicat	time:centile	centile:medication
0.571	0.302	0.388	0.000	0.333	0.878
0.571	0.170	0.514	0.001	0.226	0.878
0.571	0.013	0.080	0.001	0.333	0.117
0.670	0.092	0.999	0.001	0.895	0.449
0.571	0.025	0.080	0.001	0.226	0.158
0.571	0.002	0.001	0.000	0.908	0.050
0.571	0.000	0.060	0.000	0.008	0.000
0.571	0.005	0.165	0.000	0.017	0.057
0.571	0.122	0.153	0.000	0.005	0.637
0.670	0.269	0.987	0.001	0.182	0.980
0.902	0.193	0.514	0.001	0.368	0.654
0.571	0.100	0.514	0.001	0.384	0.637
0.571	0.012	0.060	0.001	0.164	0.104
0.810	0.158	0.306	0.000	0.157	0.012
0.594	0.000	0.287	0.000	0.009	0.000
0.571	0.104	0.153	0.001	0.333	0.637
0.670	0.364	0.987	0.001	0.489	0.980
0.670	0.010	0.809	0.001	0.024	0.081
0.940	0.698	0.287	0.000	0.335	0.192
0.571	0.000	0.000	0.000	0.004	0.000
0.571	0.690	0.361	0.000	0.112	0.620
0.574	0.481	0.514	0.001	0.178	0.880
0.571	0.000	0.182	0.000	0.004	0.001
0.670	0.528	0.999	0.000	0.399	0.081
0.699	0.028	0.999	0.001	0.226	0.205
0.571	0.010	0.287	0.000	0.819	0.081
0.785	0.012	0.999	0.001	0.162	0.081
0.571	0.017	0.153	0.001	0.039	0.168
0.571	0.023	0.182	0.001	0.333	0.219
0.696	0.000	0.768	0.000	0.039	0.000
0.571	0.481	0.514	0.001	0.338	0.580
0.949	0.010	0.388	0.001	0.399	0.078
0.571	0.273	0.193	0.001	0.178	0.980
0.670	0.180	0.999	0.001	0.730	0.803

cortical area	time	age inclusi	sex	etiv	cpz
bankssts	-27.60	-1.28	-0.69	-0.21	-26.57
caudalanteriorcingulate	-22.25	-1.62	-0.82	-1.36	-23.41
caudalmiddlefrontal	-22.37	-1.62	-0.80	-1.12	-24.71
cuneus	-23.53	-0.97	-1.20	0.93	-24.52
entorhinal	-19.79	-1.60	-0.50	-2.76	-19.13
frontalpole	-27.94	-1.89	-0.44	-2.22	-17.32
fusiform	-31.67	-1.67	-0.78	-1.70	-13.18
inferiorparietal	-24.76	-1.15	-0.89	-0.45	-25.79
inferiortemporal	-23.30	-1.73	-0.55	-3.02	-26.32
insula	-27.00	-1.74	-0.78	-0.27	-32.94
isthmuscingulate	-18.93	-1.57	-0.78	0.07	-28.19
lateraloccipital	-28.57	-1.21	-1.14	-0.51	-23.82
lateralorbitofrontal	-23.25	-1.63	-0.61	-2.37	-28.41
lingual	-28.34	-1.41	-0.94	-0.40	-19.64
medialorbitofrontal	-24.24	-1.56	-0.78	-2.07	-25.68
middletemporal	-27.85	-1.56	-0.81	-1.14	-30.59
paracentral	-25.08	-0.88	-1.00	1.06	-17.51
parahippocampal	-30.69	-1.66	-0.82	-1.94	-19.88
parsopercularis	-24.97	-1.51	-0.85	0.03	-26.40
parsorbitalis	-23.49	-1.73	-0.71	-2.17	-23.17
parstriangularis	-23.68	-1.76	-0.63	-1.76	-29.11
pericalcarine	-22.73	-1.63	-0.95	-0.62	-23.28
postcentral	-27.36	-0.98	-0.97	0.85	-16.92
posteriorcingulate	-25.59	-1.18	-0.91	0.04	-26.39
precentral	-25.45	-1.11	-0.95	0.15	-20.18
precuneus	-21.18	-1.03	-1.25	1.22	-23.85
rostralanteriorcingulate	-18.86	-1.44	-0.88	-2.34	-28.67
rostralmiddlefrontal	-26.94	-1.91	-0.73	-2.59	-28.99
superiorfrontal	-28.45	-1.57	-0.90	-0.49	-25.65
superiorparietal	-29.06	-0.64	-1.77	2.54	-14.23
superiortemporal	-28.95	-1.48	-0.85	-0.54	-29.97
supramarginal	-31.63	-1.41	-1.15	1.78	-19.82
temporalpole	-25.71	-1.56	-1.17	-0.97	-26.77
transversetemporal	-33.23	-1.38	-0.74	-0.54	-24.01

T Values				
centile	time:medication	time:centile	centile:medication	time
-2.73	40.72	2.59	3.05	0.0000
-0.94	38.91	-4.00	2.45	0.0000
-1.56	41.82	-4.41	5.19	0.0000
-7.13	37.63	-4.00	5.51	0.0000
5.34	41.81	-4.60	3.36	0.0000
5.13	42.44	2.87	-7.15	0.0000
2.46	43.14	7.58	-11.42	0.0000
-2.24	40.47	-0.02	4.52	0.0000
3.22	41.45	-2.93	5.78	0.0000
-3.66	41.02	-7.63	10.77	0.0000
-4.43	41.03	-6.33	11.86	0.0000
-2.65	40.44	-1.62	0.47	0.0000
1.33	42.52	-4.82	8.69	0.0000
-2.57	40.57	1.88	-1.30	0.0000
0.51	40.38	-6.52	3.47	0.0000
-0.83	41.80	-1.28	8.47	0.0000
-5.21	41.83	3.75	-1.99	0.0000
2.28	41.18	2.64	-3.87	0.0000
-4.83	37.05	-4.49	3.71	0.0000
1.87	40.68	-3.70	3.70	0.0000
-0.18	40.95	-5.39	8.47	0.0000
-3.77	39.89	-1.69	5.74	0.0000
-4.93	39.38	2.43	-5.08	0.0000
-3.32	39.30	-0.70	4.74	0.0000
-3.71	41.81	1.51	-1.17	0.0000
-5.55	39.86	-2.48	4.77	0.0000
0.63	40.68	-8.40	10.40	0.0000
1.80	41.64	-4.72	5.64	0.0000
-2.26	39.64	-1.55	1.77	0.0000
-7.53	39.12	7.75	-9.15	0.0000
-2.35	40.71	-1.74	4.54	0.0000
-5.99	41.17	3.71	-2.96	0.0000
-2.86	38.08	-3.54	5.75	0.0000
-2.29	39.31	4.94	1.32	0.0000

p Values						
age inclusion	sex	etiv	cpz	centile	time:medicat	time:centile
0.2730	0.5715	0.9460	0.0000	0.0128	0.0000	0.0143
0.2256	0.5715	0.4216	0.0000	0.3963	0.0000	0.0001
0.2256	0.5715	0.5279	0.0000	0.1444	0.0000	0.0000
0.3518	0.5715	0.6015	0.0000	0.0000	5.945731369	0.0001
0.2256	0.6355	0.0955	0.0000	0.0000	0.0000	0.0000
0.2256	0.6616	0.1267	0.0000	0.0000	0.0000	0.0066
0.2256	0.5715	0.2309	0.0000	0.0236	0.0000	0.0000
0.3038	0.5715	0.8220	0.0000	0.0340	0.0000	0.9813
0.2256	0.6154	0.0869	0.0000	0.0029	0.0000	0.0058
0.2256	0.5715	0.9267	0.0000	0.0007	0.0000	0.0000
0.2256	0.5715	0.9757	0.0000	0.0000	0.0000	0.0000
0.2975	0.5715	0.8151	0.0000	0.0151	0.0000	0.1231
0.2256	0.5914	0.1087	0.0000	0.2156	0.0000	0.0000
0.2365	0.5715	0.8358	0.0000	0.0183	0.0000	0.0779
0.2256	0.5715	0.1437	0.0000	0.6273	0.0000	0.0000
0.2256	0.5715	0.5279	0.0000	0.4440	0.0000	0.2130
0.3894	0.5715	0.5428	0.0000	0.0000	0.0000	0.0004
0.2256	0.5715	0.1771	0.0000	0.0336	0.0000	0.0130
0.2361	0.5715	0.9757	0.0000	0.0000	0.0000	0.0000
0.2256	0.5715	0.1277	0.0000	0.0805	0.0000	0.0004
0.2256	0.5914	0.2231	0.0000	0.8542	0.0000	0.0000
0.2256	0.5715	0.8151	0.0000	0.0005	0.0000	0.1095
0.3518	0.5715	0.6366	0.0000	0.0000	0.0000	0.0207
0.2986	0.5715	0.9757	0.0000	0.0022	0.0000	0.4985
0.3134	0.5715	0.9651	0.0000	0.0006	0.0000	0.1432
0.3443	0.5715	0.5007	0.0000	0.0000	0.0000	0.0184
0.2365	0.5715	0.1087	0.0000	0.5600	0.0000	0.0000
0.2256	0.5715	0.0955	0.0000	0.0899	0.0000	0.0000
0.2256	0.5715	0.8151	0.0000	0.0340	0.0000	0.1375
0.5247	0.5715	0.0955	0.0000	0.0000	0.0000	0.0000
0.2361	0.5715	0.8151	0.0000	0.0300	0.0000	0.1025
0.2365	0.5715	0.2231	0.0000	0.0000	0.0000	0.0004
0.2256	0.5715	0.5920	0.0000	0.0091	2.695006557	0.0007
0.2366	0.5715	0.8151	0.0000	0.0336	0.0000	0.0000

centile:medication
0.0030
0.0173
0.0000
0.0000
0.0011
0.0000
0.0000
0.0000
0.0000
0.0000
0.0000
0.6372
0.0000
0.2042
0.0007
0.0000
0.0543
0.0002
0.0003
0.0003
0.0000
0.0000
0.0000
0.0000
0.2482
0.0000
0.0000
0.0000
0.0876
0.0000
0.0000
0.0038
0.0000
0.2032

	diff	lwr	upr	p.adj
TURKEY COGNITION				
idiotypic-heteromodal	-0.08	-0.18	0.02	0.15
paralimbic-heteromodal	0.01	-0.08	0.09	1.00
unimodal-heteromodal	0.02	-0.07	0.12	0.91
paralimbic-idiotypic	0.09	-0.01	0.19	0.10
unimodal-idiotypic	0.10	0.00	0.21	0.05
unimodal-paralimbic	0.02	-0.08	0.11	0.97
TURKEY COGNITION Psychosis				
idiotypic-heteromodal	-0.44	-1.71	0.82	0.77
paralimbic-heteromodal	-0.10	-1.19	1.00	1.00
unimodal-heteromodal	-0.25	-1.41	0.91	0.93
paralimbic-idiotypic	0.35	-0.91	1.61	0.88
unimodal-idiotypic	0.19	-1.13	1.51	0.98
unimodal-paralimbic	-0.16	-1.32	1.00	0.98
TURKEY SAPS				
idiotypic-heteromodal	1.57	-1.52	4.66	0.52
paralimbic-heteromodal	-2.51	-5.18	0.17	0.07
unimodal-heteromodal	0.19	-2.65	3.02	1.00
paralimbic-idiotypic	-4.08	-7.17	-0.99	0.01
unimodal-idiotypic	-1.39	-4.62	1.84	0.65
unimodal-paralimbic	2.69	-0.15	5.53	0.07
TURKEY SANS				
idiotypic-heteromodal	-2.48	0.22	0.13	0.11
paralimbic-heteromodal	-1.85	0.49	0.41	0.42
unimodal-heteromodal	-1.79	0.70	0.64	0.62
paralimbic-idiotypic	-0.90	1.80	0.80	0.75
unimodal-idiotypic	-0.83	2.00	0.68	0.65
unimodal-paralimbic	-1.11	1.38	0.99	0.99
TURKEY BPRS				
idiotypic-heteromodal	-2.78	-7.09	1.54	0.32
paralimbic-heteromodal	0.71	-3.03	4.45	0.95
unimodal-heteromodal	0.30	-3.66	4.26	1.00
paralimbic-idiotypic	3.49	-0.83	7.80	0.15
unimodal-idiotypic	3.08	-1.43	7.59	0.27

unimodal-paralimbic	-0.41	-4.37	3.55	0.99

Dimensional Reduction analysis of clinic scales Using PCA

Cognition Subscales: Principal Components Analysis	PC1	PC2
Global_Cognitive_Functioning_Verbal_Memory	0.3713869	-0.399849
Global_Cognitive_Functioning_Visual_Memory	0.3813908	-0.230313
Global_Cognitive_Functioning_Processing_Speed	0.4087527	0.0315755
Global_Cognitive_Functioning_Working_Memory	0.3381386	-0.531875
Global_Cognitive_Functioning_Executive_Function	0.3978971	0.0873606
Global_Cognitive_Functioning_Motor_Dexterity	0.3712996	0.5446113
Global_Cognitive_Functioning_Attention	0.3728416	0.4460448

total explained variance PC1 :45,3%

SAPS Subscales: Principal Components Analysis	PC1	PC2
SAPS_Global_rating_of_positive_formal_thought_disorder	0.3642406	0.8920635
SAPS_Global_rating_of_bizzare_behavior	0.5657431	-0.07589
SAPS_Global_rating_of_delusions	0.5683927	-0.145306
SAPS_Global_rating_of_hallucinations	0.4734904	-0.421129

total explained variance PC1 : 65,5%

SANS Subscales: Principal Components Analysis	PC1	PC2
SANS_Global_Rating_of_Affective_Flattening	0.5086713	0.0639616
SANS_Global_Rating_of_Alogia	0.4873125	-0.088922
SANS_Global_Rating_of_Avolition_Apathy	0.4740683	0.2462151
SANS_Global_Rating_of_Anhedonia_Asociality	0.4723193	0.2424832
SANS_Global_Rating_of_Attention	0.2365455	-0.931977

total explained variance PC1 : 60,5%

BPRS Subscales: Principal Components Analysis	PC1	PC2
BPRS_bprsom	-0.171303	0.094871
BPRS_bprans	-0.19449	-0.01222
BPRS_bprdep	-0.123764	0.2792029
BPRS_bprcul	-0.114948	0.1949886
BPRS_bprhos	-0.285633	-0.081394
BPRS_bprsus	-0.306652	0.0763201
BPRS_bprpen	-0.319277	0.0579488
BPRS_bprgra	-0.11684	-0.189411
BPRS_bpralu	-0.243462	0.0906774
BPRS_bprori	-0.076086	-0.049708
BPRS_bprcon	-0.234168	-0.149713
BPRS_bprexc	-0.248089	-0.265081
BPRS_bprlen	-0.050185	0.4245484
BPRS_bpremb	-0.060313	0.4383074
BPRS_bprten	-0.266931	-0.097153
BPRS_bprpos	-0.195805	-0.032672
BPRS_bprcoo	-0.269715	0.0096991
BPRS_bpremo	-0.183706	0.3201454
BPRS_bprsui	-0.066174	0.1867782
BPRS_bprcui	-0.153656	0.1956501
BPRS_bprext	-0.329532	0.0023198
BPRS_bprani	-0.123272	-0.248462
BPRS_bpract	-0.180983	-0.273742
BPRS_bprdis	-0.157697	-0.156618

PC3	PC4	PC5	PC6	PC7
-0.579926	0.0130289	-0.184055	-0.308534	-0.486456
0.043421	0.7901455	0.1901522	0.007926	0.372909
-0.419338	-0.466474	0.0224627	0.2248082	0.622445
0.5348805	-0.230267	-0.186926	0.4496243	-0.162936
0.3577289	-0.286903	0.5553612	-0.55854	-0.058052
-0.116067	0.1293753	0.261608	0.5156177	-0.448358
0.241697	0.07634	-0.719483	-0.272424	0.0769289

Cognition PC1

Global_Co
Global_Cognitive
Global_Cognitive_Fu
Global_Cognitive_F
Global_Cognitive_Fu
Global_Cognitive
Global_Cognitive

PC3	PC4
0.2657191	-0.030738
-0.440582	0.6928661
-0.374386	-0.718088
0.7714379	0.0578003

SAPS PC1 des

SAPS_Global_rating_of_hallucinations
SAPS_Global_rating_of_delusions
SAPS_Global_rating_of_bizzare_behavio
SAPS_Global_rating_of_positive_formal_thou ght_disorder

PC3	PC4	PC5
-0.407919	0.1566037	-0.739081
-0.588326	-0.099238	0.6313823
0.4307225	-0.726072	-0.04399
0.4803296	0.6621527	0.2212548
0.2669015	0.0006782	-0.06502

SANS PC

SANS_Global_Rating_of_Attent
SANS_Global_Rating_of_Anhedonia_Asocial

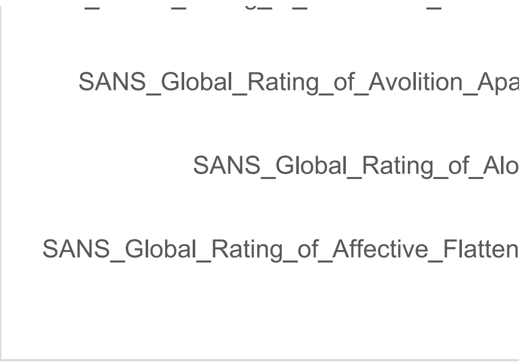

PC3	PC4	PC5	PC6	PC7	PC8	PC9
-0.07668	-0.119093	-0.020404	-0.66901	0.2516705	0.1232375	-0.076009
-0.265438	0.1217031	-0.120745	-0.154858	0.5332697	-0.177306	0.0615722
-0.264428	0.3472469	-0.059108	0.0181283	0.173398	0.0648918	0.1721241
-0.155283	0.4077504	-0.260373	-0.002525	-0.089281	0.3291751	0.3308789
-0.123293	-0.198729	0.10575	0.1969122	-0.050234	0.0036134	0.1657589
-0.219365	-0.015064	0.083115	0.0150624	-0.17402	-0.180932	-0.074796
-0.164441	0.0253204	0.1214028	-0.066068	-0.196292	-0.168275	-0.124781
0.2496915	0.3361788	0.3920759	-0.217458	-0.238226	0.0190828	0.2163282
-0.057921	-0.109489	0.1157744	-0.226588	-0.16429	-0.145389	-0.33945
0.1300423	0.0486162	-0.660427	-0.153139	-0.387345	-0.361987	0.2364253
0.2123799	0.0673287	-0.20088	-0.04641	0.011804	0.0727875	-0.054182
-0.00293	-0.058929	0.0041639	0.1831717	0.1707136	-0.026446	0.1986272
0.3149219	0.0478846	0.0224776	0.0342349	0.121559	-0.226361	0.0496833
0.3065437	0.0145981	0.0676679	0.0919067	0.1069332	-0.212356	0.0272957
-0.148444	-0.160799	0.0231556	0.2666147	0.1107247	-0.020055	0.1835341
0.1703668	-0.181575	-0.226709	-0.156421	-0.150087	0.5210553	-0.132291
0.1286285	-0.239259	-0.016084	0.1767784	-0.084373	0.1282901	0.1292624
0.29298	-0.118419	0.0344598	0.0845436	-0.004775	-0.05715	0.0429659
-0.195901	0.3851274	-0.001977	0.3052249	-0.236238	0.1790009	-0.446623
0.1995057	-0.078344	0.0986362	0.0286194	0.1238664	0.4325291	0.0882088
-0.135962	-0.048098	0.0872642	-0.039281	-0.148796	-0.081539	-0.033341
0.2697651	0.4035159	0.2714967	-0.165641	0.0020967	-0.061599	0.0410585
0.2073788	0.1862651	-0.044424	0.2064662	0.2073798	-0.043335	-0.044927
0.2291432	0.1467509	-0.292796	0.1334098	0.2746048	-0.042483	-0.507298

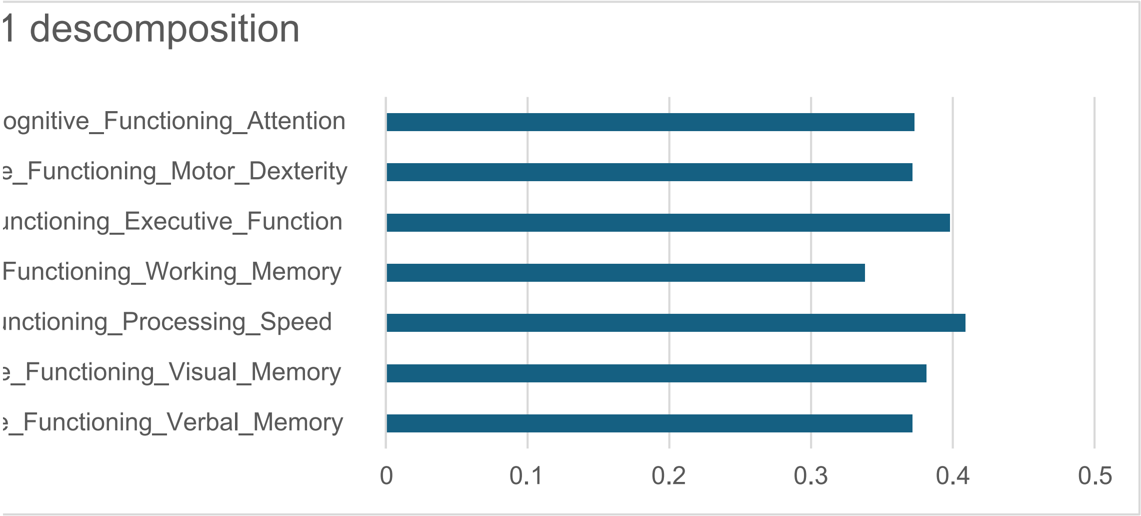
1 descomposition
ognitive_Functioning_Attention
e_Functioning_Motor_Dexterity
ınctioning_Executive_Function
Functioning_Working_Memory
ınctioning_Processing_Speed
e_Functioning_Visual_Memory
e_Functioning_Verbal_Memory
0
0.1
0.2
0.3
0.4
0.5

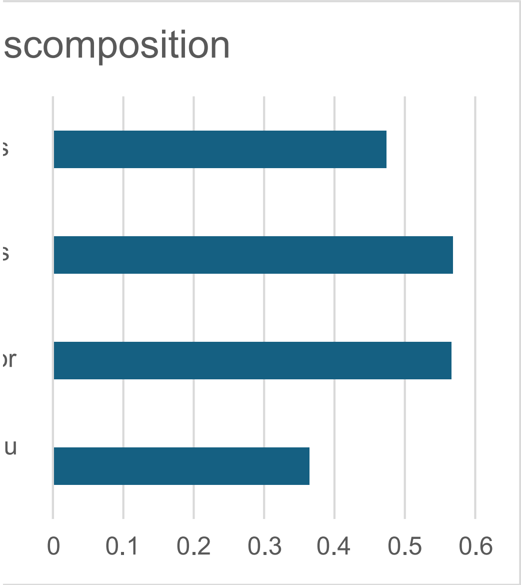
scomposition
0
0.1
0.2
0.3
0.4
0.5
0.6

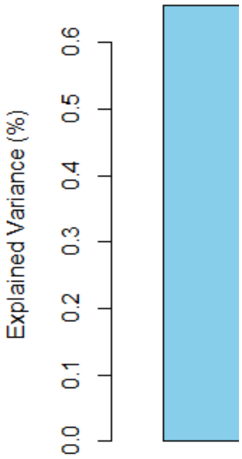
Explained Variance (%)
0.0
0.1
0.2
0.3
0.4
0.5
0.6

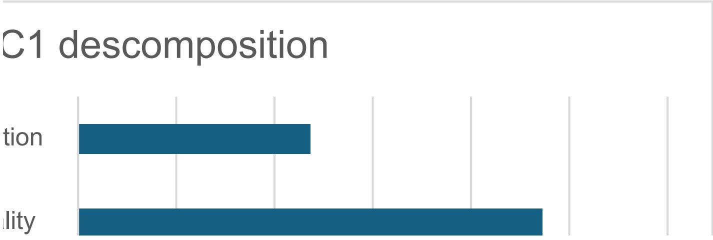
C1 descomposition
tion
lity

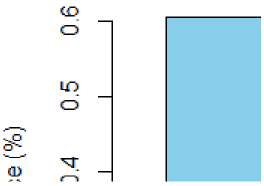
0.5
0.6

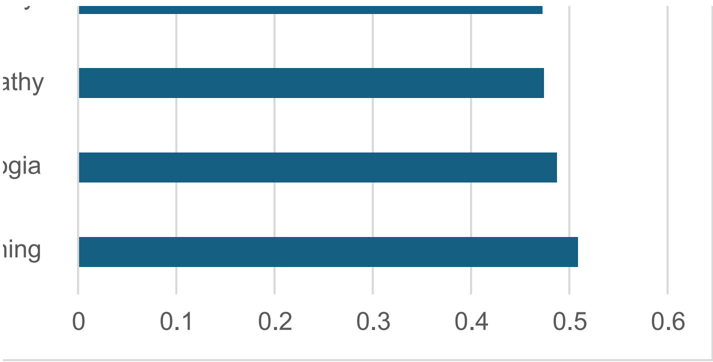

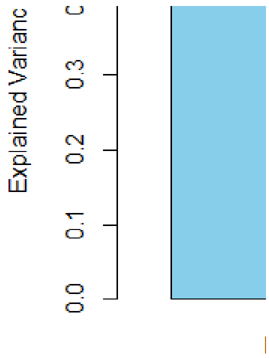

PC10	PC11	PC12	PC13	PC14	PC15	PC16
-0.029454	-0.360212	0.3284813	0.1423843	0.042533	-0.274974	0.0046169
0.0426739	-0.157727	-0.403624	-0.188811	0.0543576	0.1376391	-0.192438
-0.006737	0.1190109	-0.126887	0.1560253	-0.186343	0.132849	0.6936885
0.1917726	0.2996468	0.3718824	0.0719818	0.1491402	-0.004769	-0.373737
-0.001561	-0.069481	0.1156886	0.2187284	0.1058243	-0.029541	-0.01322
0.0034327	0.1658279	-0.066433	-0.120992	-0.224153	-0.203647	-0.131988
-0.01572	0.1535709	-0.057746	-0.149682	-0.160221	-0.156766	-0.123487
0.1066022	-0.060362	-0.297324	-0.051333	0.350707	-0.006303	0.0616545
-0.115444	0.2030395	0.1885627	0.2237315	0.264253	0.6561757	0.0663094
-0.28457	-0.188636	-0.164207	0.1575912	0.0224815	0.0133528	0.0097456
-0.052019	0.0925877	0.2436348	-0.693852	0.1967553	-0.012103	0.2856696
-0.065014	-0.167652	0.2090951	-0.095586	0.0890531	0.1721096	-0.023883
0.1222581	-0.063304	-0.020308	0.0344884	-0.009758	0.0699025	-0.221144
0.095779	-0.036898	0.0411689	-0.065465	0.0281232	0.0500228	-0.107617
0.1445772	-0.090862	-0.165377	0.1138546	0.3516221	0.0445836	-0.080145
0.40527	-0.061694	-0.360196	-0.028563	-0.268799	0.2865078	-0.090661
0.1209628	-0.158336	0.0768481	0.252923	-0.034821	-0.224728	0.2514731
0.1383474	-0.093061	0.0578913	-0.117865	-0.021505	-0.05828	0.2026819
-0.11208	-0.593968	0.0032491	-0.055181	0.1296309	-0.021543	-0.048784
-0.750862	0.1493495	-0.237252	0.027713	0.0431505	-0.044736	-0.134881
-0.041058	0.137343	-0.055178	-0.075795	-0.209809	-0.177739	-0.039027
0.0201053	-0.051506	0.0272099	0.251967	-0.198582	-0.063545	-0.025123
-0.130318	-0.10133	0.2422669	0.0564449	-0.492809	0.2666447	-0.122687
0.0956921	0.3321546	-0.111475	0.2970284	0.2602841	-0.325182	0.0390023

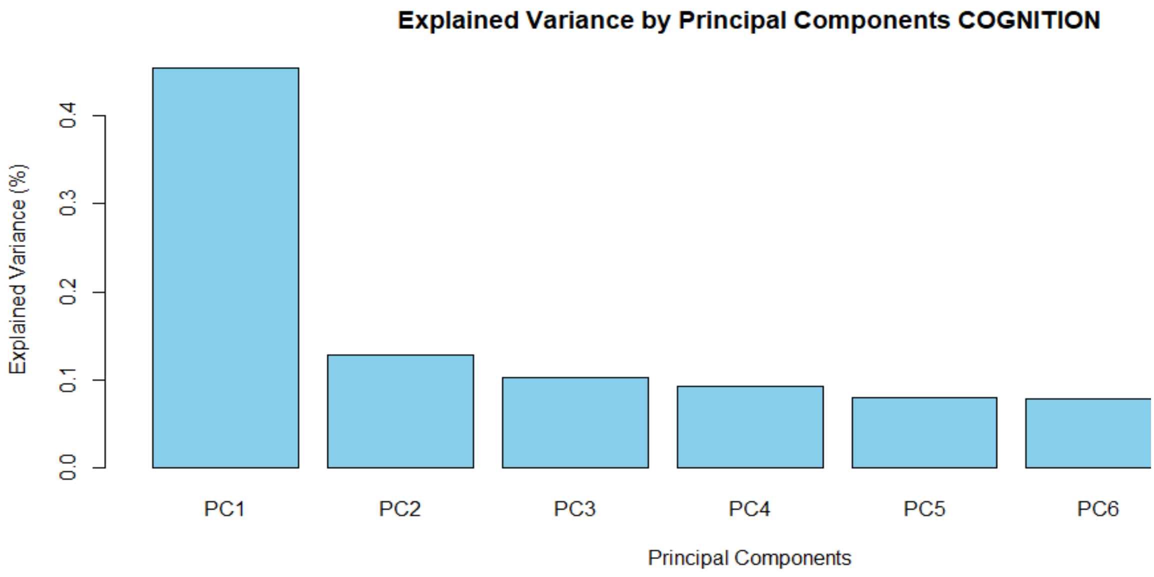
Explained Variance by Principal Components COGNITION
Explained Variance (%)
0.0
0.1
0.2
0.3
0.4
PC1
PC2
PC3
PC4
PC5
PC6
Principal Components

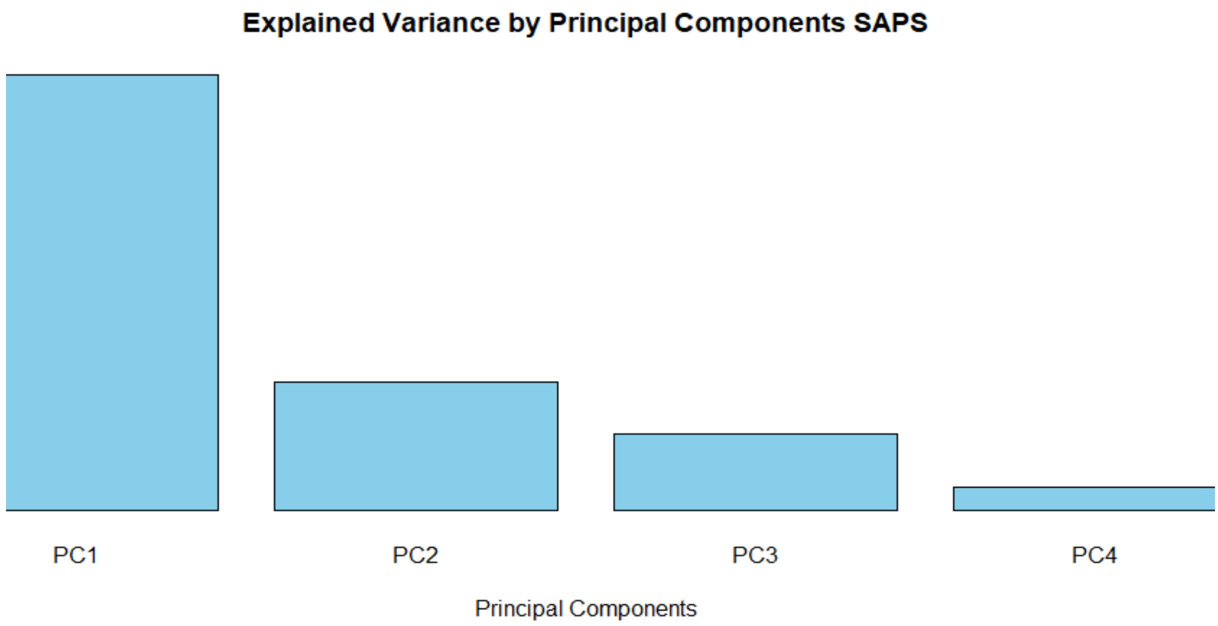
Explained Variance by Principal Components SAPS
PC1
PC2
PC3
PC4
Principal Components

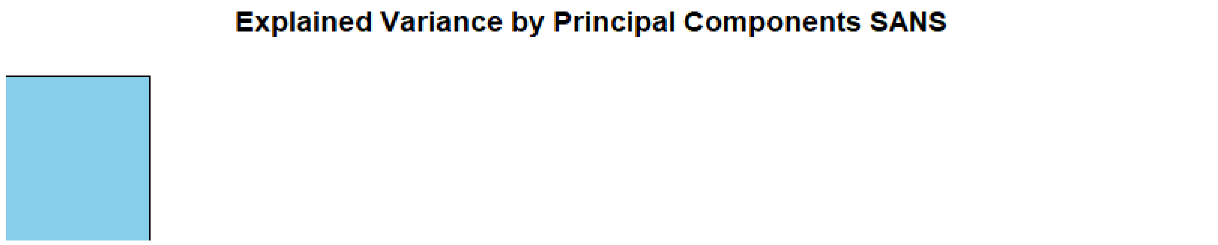
Explained Variance by Principal Components SANS

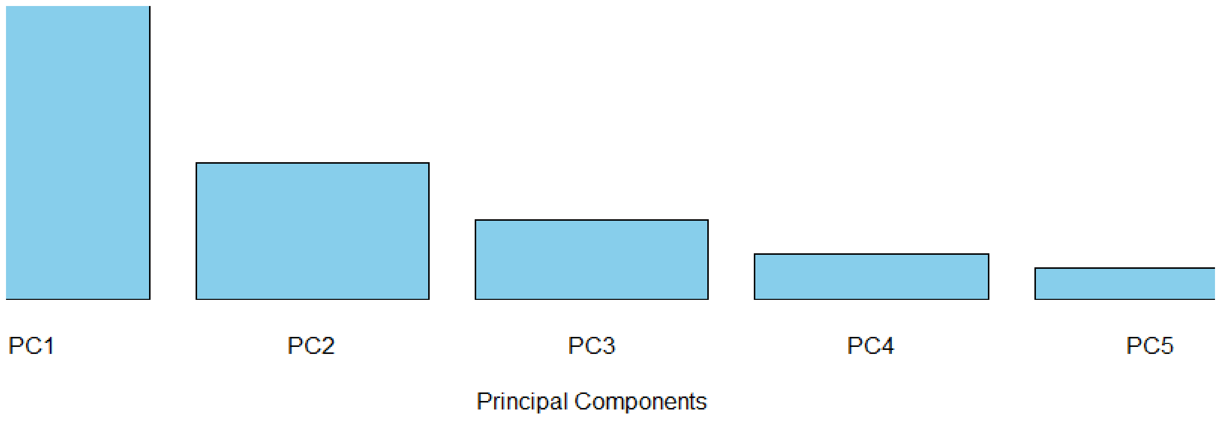

PC17	PC18	PC19	PC20	PC21	PC22	PC23
-0.213523	-0.121643	-0.10466	-0.005664	-0.002819	-0.031013	-0.014079
0.4481933	0.0340323	0.1059143	0.1265616	0.0636208	-0.022746	0.0236795
-0.230858	-0.036807	0.0455154	-0.045503	-0.006037	-0.008781	-0.020625
0.1574854	-0.019159	-0.05394	0.0368477	-0.049245	0.0047952	0.0301071
-0.169512	0.098635	0.4091497	0.6164511	0.3192168	-0.039482	-0.068121
-0.052526	-0.05826	-0.008548	-0.070619	-0.074207	-0.256306	-0.525714
-0.052026	-0.047645	-0.040246	-0.087513	-0.068848	-0.155576	0.0212807
-0.032485	-0.46601	0.0160218	0.1064181	-0.064906	-0.011795	0.0023741
0.1910589	0.0178847	-0.027605	-0.05219	0.00941	0.0137409	-0.014137
-0.043866	0.0305974	-0.028155	0.0307734	-0.020584	-0.037964	0.0166234
-0.027165	0.1320082	0.0477802	-0.060951	0.3456493	0.0243734	-0.072034
-0.178919	0.054845	0.3332032	-0.236424	-0.660615	-0.003242	0.0120046
-0.182366	-0.074079	0.2487501	-0.223165	0.1660932	0.5279657	-0.32344
-0.152181	0.0131491	0.0982091	-0.057944	0.0802185	-0.586574	0.4477815
-0.297232	0.1429899	-0.588516	-0.23992	0.196085	0.0276946	0.0007829
-0.168675	0.0805204	0.0988923	0.0080104	-0.034945	-0.063409	-0.004664
0.5697689	-0.192709	0.1325827	-0.382717	0.1567333	-0.074601	-0.002557
0.2348897	0.1556783	-0.397188	0.4705058	-0.4144	0.1347983	-0.105034
0.0170547	0.0390005	0.0305767	-0.003868	-0.001622	0.0365547	0.0208548
0.0115483	0.0413731	-0.026324	-0.021955	-0.014812	-0.02382	-0.048086
-0.028325	0.0059594	0.0257863	-0.009547	-0.006642	0.4994409	0.6241409
0.0867961	0.6633275	-0.010484	-0.104635	0.0837957	-0.040708	-0.044865
-0.030443	-0.428141	-0.27914	0.1319849	0.1584331	-0.015585	0.0033948
-0.079306	-0.086806	0.0729509	0.075277	-0.135471	-0.002554	0.010949

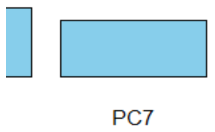
PC7

PC24
-0.022814
-0.000906
0.0306266
-0.002529
0.0696024
-0.511424
0.7789628
-0.056081
-0.05759
-0.000108
-0.016789
0.0040779
0.0989675
-0.145015
-0.002903
-0.00015
0.0328944
0.0311924
-0.030775
0.0093166
-0.290589
-0.012822
0.0060276
-0.005303

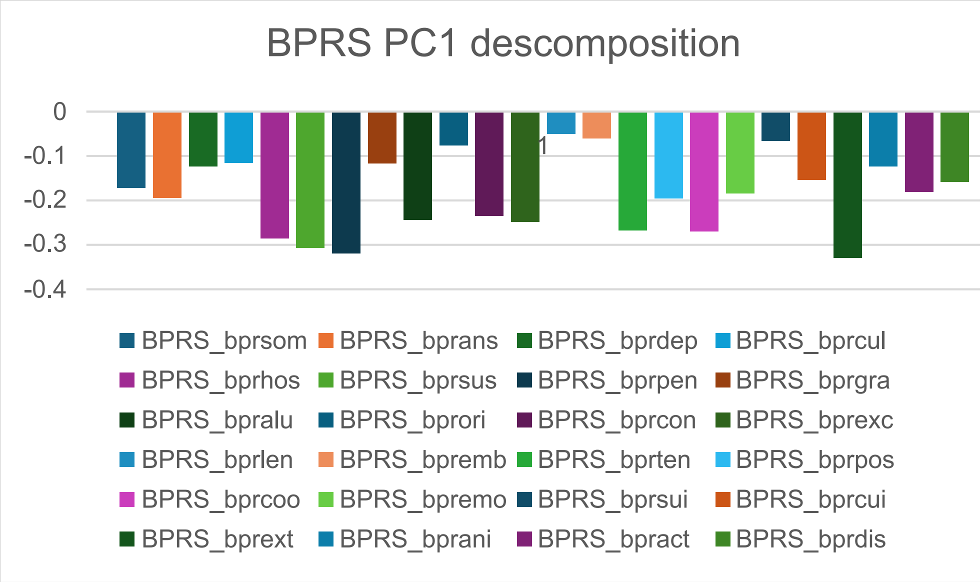

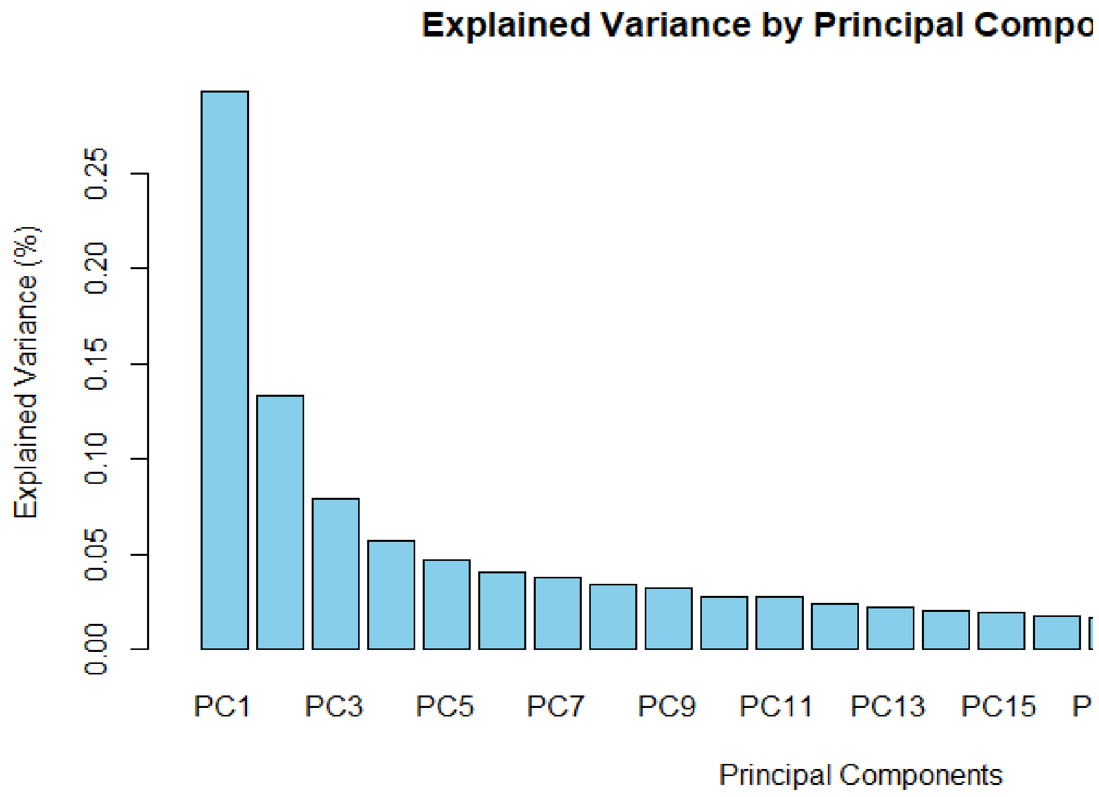
Explained Variance by Principal Compo
Explained Variance (%)
0.00
0.05
0.10
0.15
0.20
0.25
PC1
PC3
PC5
PC7
PC9
PC11
PC13
PC15
P
Principal Components

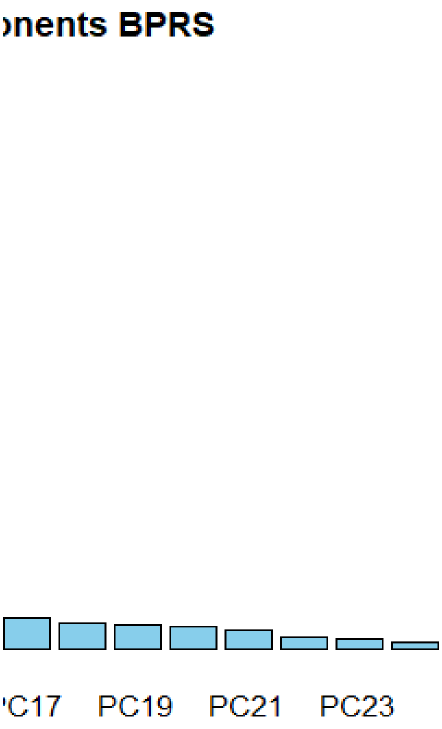
nents BPRS
C17
PC19
PC21
PC23

route	Time	mean
ute 1 (via centi	0	-0.005
	1	-0.002
	3	0.002
	5	0.007
	10	0.018
2 (direct, no ce	0	0.083
	1	-0.047
	3	-0.305
	5	-0.564
	10	-1.210
Total	0	0.078
	1	-0.049
	3	-0.303
	5	-0.557
	10	-1.192

Medication- cognition Mediation Analysis	
Lower 2.5% CI (lower bound)	Upper 95% CI (upper bound)
-0.013	0.001
-0.010	0.004
-0.008	0.014
-0.008	0.025
-0.009	0.056
0.012	0.152
-0.139	0.045
-0.496	-0.116
-0.869	-0.262
-1.812	-0.619
0.008	0.147
-0.140	0.041
-0.493	-0.116
-0.859	-0.259
-1.791	-0.604

Probability greater than 0 (Pr(β>0))	Bayesian p-value (MCMC-based)
0.050	0.101
0.211	0.422
0.654	0.693
0.811	0.378
0.895	0.211
0.990	0.020
0.155	0.310
0.001	0.001
0.000	0.000
0.000	0.000
0.987	0.026
0.143	0.286
0.001	0.001
0.000	0.000
0.000	0.000

Model	Multiple R squared
Baseline (GMV)	0.43
Baseline (WMV)	0.36
	R marginal
Longitudinal structural	0.42
Cognition longitudinal	0.26
SAPS longitudinal	0.93
SANS longitudinal	0.04
BPRS longitudinal	0.64

Adjusted R squared	p-value
0.42	$<10^{-15}$
0.36	$<10^{-15}$
R conditional	
0.94	
0.76	
0.95	
0.90	
0.97	
